# An electrically driven single-atom “flip-flop” qubit

**DOI:** 10.1126/sciadv.add9408

**Published:** 2023-02-10

**Authors:** Rostyslav Savytskyy, Tim Botzem, Irene Fernandez de Fuentes, Benjamin Joecker, Jarryd J. Pla, Fay E. Hudson, Kohei M. Itoh, Alexander M. Jakob, Brett C. Johnson, David N. Jamieson, Andrew S. Dzurak, Andrea Morello

**Affiliations:** ^1^ School of Electrical Engineering and Telecommunications, UNSW Sydney, Sydney, NSW 2052, Australia.; ^2^ School of Fundamental Science and Technology, Keio University, Kohoku-ku, Yokohama, Japan.; ^3^ School of Physics, University of Melbourne, Melbourne, VIC 3010, Australia.

## Abstract

The spins of atoms and atom-like systems are among the most coherent objects in which to store quantum information. However, the need to address them using oscillating magnetic fields hinders their integration with quantum electronic devices. Here, we circumvent this hurdle by operating a single-atom “flip-flop” qubit in silicon, where quantum information is encoded in the electron-nuclear states of a phosphorus donor. The qubit is controlled using local electric fields at microwave frequencies, produced within a metal-oxide-semiconductor device. The electrical drive is mediated by the modulation of the electron-nuclear hyperfine coupling, a method that can be extended to many other atomic and molecular systems and to the hyperpolarization of nuclear spin ensembles. These results pave the way to the construction of solid-state quantum processors where dense arrays of atoms can be controlled using only local electric fields.

## Teaser

The combined electron-nuclear spins of a single donor atom in silicon are coherently controlled using electric fields.

## INTRODUCTION

A century ago, understanding the electronic structure and optical spectra of atoms was one of the first successes of the emerging theory of quantum mechanics. Today, atoms and atom-like systems constitute the backbone of coherent quantum technologies (*1*), providing well-defined states to encode quantum information, act as quantum sensors, or interface between light and matter. Their spin degree of freedom (*2*) is most often used in quantum information processing because of its long coherence time, which can stretch to hours for atomic nuclei (*3*, *4*).

Moving from proof-of-principle demonstrations to functional quantum processors requires strategies to engineer multiqubit interactions and to integrate atom-based spin qubits with control and interfacing electronics (*5*). There, the necessity to apply oscillating magnetic fields to control the atom’s spin poses challenging engineering problems. Magnetic fields oscillating at radio frequency (rf; for nuclear spins) or microwaves (MWs; for electron spins) cannot be localized or shielded at the nanoscale, and their delivery is accompanied by large power dissipation, difficult to reconcile with the cryogenic operation of most quantum devices. Therefore, many spin-based quantum processors based on “artificial atoms” (quantum dots) rely instead on electric fields for qubit control, through a technique called electric dipole spin resonance (EDSR) (*6*, *7*).

The longer coherence time of natural atoms and atom-like systems is accompanied by a reduced sensitivity to electric fields, making EDSR more challenging. EDSR was demonstrated in ensembles of color centers in silicon carbide (*8*) for electron spins and ensembles of donors in silicon for nuclear spins (*9*). At the single-atom level, coherent electrical control was demonstrated in scanning tunneling microscope experiments (*10*), single-atom molecular magnets (*11*), and high-spin donor nuclei (*12*). Still lacking is a method to perform EDSR with single atoms, at MW frequencies, in a platform that enables easy integration with control electronics and large-scale manufacturing.

Here, we report the coherent electrical control of a spin qubit formed by the antiparallel states of the electron and nuclear spins of a single ^31^P atom in silicon, thus called the “flip-flop” qubit (*13*). The MW electric field produced by a local gate electrode induces coherent quantum transitions between the flip-flop states via the modulation of the electron-nuclear hyperfine interaction *A*, which depends on the precise shape of the electron charge distribution. Local control of *A* with baseband electrical pulses was already suggested in the seminal Kane proposal (*14*) as a way to select a specific qubit to be operated within a global rf magnetic field (*15*). Here, instead, we control the qubit directly with local MW electric signals.

The definition and operation of the flip-flop qubit can be understood on the basis of the spin Hamiltonian of the ^31^P donor system. In the absence of time-dependent fields, and expressed in frequency units, it reads

$$\hat{H}=\gamma_{e}B_{0}{\hat{S}}_{z}-\gamma_{n}B_{0}{\hat{I}}_{z}+A\hat{S}\cdot \hat{I}$$ (1)

where $\hat{S}=({\hat{S}}_{x},{\hat{S}}_{y},{\hat{S}}_{z})$ and $\hat{I}=({\hat{I}}_{x},{\hat{I}}_{y},{\hat{I}}_{z})$ are the vector spin operators for the electron and nucleus, respectively. The first and second terms represent the electron and nuclear Zeeman energies created by a magnetic field **B**_0_, whose direction defines the *z* axis in the spin Hamiltonian. The electron and nuclear gyromagnetic ratios are γ_e_ ≈ 27.97 GHz/T and γ_n_ ≈ 17.25 MHz/T, respectively. For simplicity, we define γ_e_ and γ_n_ as both positive and account for their sign in the Hamiltonian definition. The third term describes the Fermi contact hyperfine interaction, which is proportional to the overlap of the electron wave function with the nucleus. For a ^31^P donor in bulk silicon, *A* = 117.53 MHz. Electric fields (*15*) and/or lattice strain (*16*) can distort the electron wave function and modify the value of *A*.

**Fig. 1.**
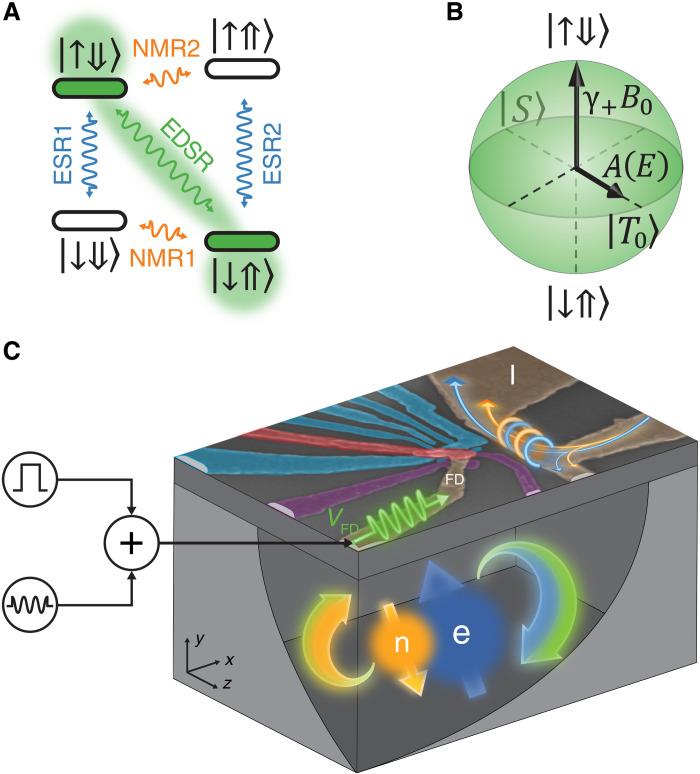
Flip-flop qubit and device layout. (**A**) Energy level diagram of ^31^P donor electron (↑,↓) and nuclear (⇑,⇓) spin states, in the presence of a static magnetic field **B**_0_ ∼ 1 T along the *z* direction. Electron spin resonance (ESR) and nuclear magnetic resonance (NMR) transitions are induced by oscillating magnetic fields. The flip-flop qubit is obtained by truncating the system to the ↓⇑ , ↑⇓ states, between which transitions are induced by EDSR. (**B**) Bloch sphere representation of the flip-flop qubit. (**C**) False-color scanning electron microscopy image of the device, comprising a single-electron transistor (SET) (cyan) to read out the electron spin, local gate electrodes (red and purple) to control the donor potential, and MW antennas (brown) for electric (left, open-circuit) and magnetic (right, short-circuit) control of the donor spins. Here and elsewhere, we use the color orange to represent properties related to the nuclear spin, blue for the electron spin, and green for the flip-flop qubit.

In the presence of a static magnetic field strong enough to ensure that γ_e_B_0_ ≫ *A*, the eigenstates of this two-spin system are approximately the tensor product of electron-nuclear spin states: ∣↓,↑〉 ⊗ ∣⇑,⇓〉 ∈ {∣↓⇑〉, ∣↓⇓〉, ∣↑⇓〉, ∣↑⇑〉}. We use here B_0_ ≈ 1 T, whereby γ_e_B_0_ ≈ 28 GHz ≫ *A* = 114.1 MHz. The energy level diagram thus takes the simple form shown in Fig. 1A.

The standard way to control the electron-nuclear spin states of the ^31^P donor is to apply an oscillating magnetic field of the form *B*_1_ cos (2πν*t*) with $B_{1}⊥B_{0}$. The resulting spin Hamiltonian term $\left(\gamma_{e}{\hat{S}}_{x}-\gamma_{n}{\hat{I}}_{x})B_{1}\cos(2\pi \nu t\right)$ has nonzero matrix elements between electron-nuclear states where the spin angular momentum changes by 1ℏ (ℏ is the reduced Planck constant). These are thus the first-order magnetically allowed spin transitions. As shown in Fig. 1A, they can be divided into two groups: electron spin resonance (ESR) (*17*), where the electron spin state changes and the nuclear spin remains fixed (ESR1, ∣↓⇓〉 ↔ ∣↑⇓〉 at frequency ν_e1_ = γ_e_B_0_ − *A*/2, and ESR2, ∣↓⇑〉 ↔ ∣↑⇑〉 at frequency ν_e2_ = γ_e_B_0_ + *A*/2), and nuclear magnetic resonance (NMR) (*18*), where the nuclear spin state changes and the electron spin remains fixed (NMR1, ∣↓⇓〉 ↔ ∣↓⇑〉 at frequency ν_n1_ = *A*/2 + γ_n_B_0_, and NMR2, ∣↑⇓〉 ↔ ∣↑⇑〉 at frequency ν_n2_ = *A*/2 − γ_n_B_0_). In our setup, the oscillating magnetic field *B*_1_ is delivered by an on-chip broadband MW antenna (see Fig. 1C), terminated by a short circuit (*19*) to maximize the magnetic component of the field produced at its tip. ESR driving signals in the ∼30 GHz range and NMR signals in the ∼30 to 70 MHz range are combined at room temperature and delivered to the donor by the same antenna (see Fig. 1C and Materials and Methods for more details).

The key aspect of our work consists in recognizing that, in the electron-nuclear Hamiltonian in Eq. 1, the hyperfine term couples directly the ∣↓⇑〉 and ∣↑⇓〉 states. Written explicitly

$$\hat{H}=\frac{1}{2}\left(\begin{array}{cccc} \gamma_{-}B_{0}+A/2 & 0 & 0 & 0\\ 0 & \gamma_{+}B_{0}-A/2 & A & 0\\ 0 & A & -\gamma_{+}B_{0}-A/2 & 0\\ 0 & 0 & 0 & -\gamma_{-}B_{0}+A/2 \end{array}\right)$$ (2)

where γ_±_ = γ_e_ ± γ_n_, and the columns of the matrix are ordered as the ∣↑⇑〉, ∣↑⇓〉, ∣↓⇑〉, and ∣↓⇓〉 states. Because the matrix is block diagonal, we can define a truncated two-dimensional subspace, i.e., a qubit, with basis states ∣↑⇓〉, ∣↓⇑〉 as

$${\hat{H}}_{\mathrm{ff}}=\frac{1}{2}\left(\begin{array}{cc} \gamma_{+}B_{0}-A/2 & A\\ A & -\gamma_{+}B_{0}-A/2 \end{array}\right)=\frac{1}{2}\left(\gamma_{+}B_{0}{\hat{\sigma }}_{z}+A{\hat{\sigma }}_{x}-\frac{A}{2}\hat{1}\right)$$ (3)

where ${\hat{\sigma }}_{x}\;\mathrm{and}\;{\hat{\sigma }}_{z}$ are Pauli matrices and $\hat{1}$ is the identity matrix. We call flip-flop qubit the two-level system described by the above Hamiltonian. Its Bloch sphere is depicted in Fig. 1B. The longitudinal Hamiltonian term is set by the sum of the electron and nuclear Zeeman energies, $\gamma_{+}B_{0}{\hat{\sigma }}_{z}$. The basis states are coupled by the term $A{\hat{\sigma }}_{x}$. In other words, within the flip-flop qubit subspace, the hyperfine interaction acts in the same way a transversal magnetic field would act on a simple spin-^1^/_2_ qubit. Therefore, modulating *A* at the frequency corresponding to the flip-flop states’ energy difference induces coherent spin transitions between the ∣↑⇓〉, ∣↓⇑〉 states. Crucially, the modulation of *A* is obtained by applying electric instead of magnetic fields, because it requires a local distortion of the electron wave function at the donor.

To operate the flip-flop qubit, we fabricated a device that, in addition to the short-circuited MW antenna used for NMR and ESR, contains a second on-chip transmission line, labeled fast donor (FD) gate, which is terminated by an open circuit to maximize the electric component of the field at its tip (Fig. 1C). By applying an oscillating voltage to the FD gate, the resulting electric field *E*_ac_ cos (2πϵ_ff_*t*) at the donor location modulates *A* via the hyperfine Stark effect. Microscopically, the electric field distorts the electron wave function and time-dependently alters its overlap with the ^31^P nucleus, on which *A* depends. Distorting the electron wave function from the spherically symmetric 1s orbital ground state results in the formation of an electric dipole (*13*), because the electron charge distribution is laterally displaced with respect to the positive nuclear charge. The resulting transitions between the flip-flop states thus constitute a form of EDSR.

The resonance frequency is simply $\epsilon_{\mathrm{ff}}=\sqrt{{\left(\gamma_{+}B_{0}\right)}^{2}+A{\left(E_{\mathrm{dc}}\right)}^{2}}$, where *E*_dc_ is the static electric field at the donor location. The flip-flop Rabi frequency $f_{\mathrm{Rabi}}^{\mathrm{ff}}$ depends on the electric polarizability of the electron wave function, which is reflected in the Stark shift of the hyperfine coupling ∂*A*(*E*)/*∂E*, yielding $f_{\mathrm{Rabi}}^{\mathrm{ff}}=[\partial A(E)/2\partial E]E_{\mathrm{ac}}$, where the factor 2 accounts for the rotating wave approximation. For a donor in bulk silicon, however, *∂A*(*E*)/*∂E* = 0 (*20*) because of the spherical symmetry of the electron wave function. A static electric field *E*_dc_ and/or lattice strain are necessary to introduce an initial wave function distortion that unlocks a nonzero linear Stark effect, accompanied by a static electric dipole. In the original flip-flop qubit proposal (*13*), it was envisaged to operate the donor in a regime where *∂A*(*E*)/*∂E* and the associated electric dipole are greatly enhanced by pulling the electron halfway between the donor and an interface quantum dot. Assuming that the donor is placed at a depth *d* ≈ 10 nm below the Si/SiO_2_ interface (see Materials and Methods), this results in a large electric dipole *e* · *d* ≈ 15 debye (*e* is the electron charge) and flip-flop Rabi frequencies up to $f_{\mathrm{Rabi}}^{\mathrm{ff}}∼10$ MHz (note that $f_{\mathrm{Rabi}}^{\mathrm{ff}}$ is, in any case, upper-bound by the maximum value of *A* ≈ 100 MHz). The same large electric dipole can be used to mediate strong (∼10 MHz), long-distance (∼200 nm) coupling between flip-flop qubits.

Unlike earlier examples of electrical drive in electron-nuclear systems (*9*, *11*), the flip-flop transition does not require an anisotropic hyperfine interaction, i.e., terms of the form *A_xz_S_z_I_x_*. Such terms are necessary when attempting to drive the nuclear spin at its own resonance frequency, while leaving the electron unchanged. Here, the electron flip-flops with the nucleus, making the flip-flop transition possible even in systems where the hyperfine interaction is purely isotropic.

The operation and readout of the flip-flop qubit is achieved by fabricating a silicon metal-oxide-semiconductor device as depicted in Fig. 1C. An ion-implanted ^31^P donor is placed under a set of electrostatic gates that control its electrochemical potential, in proximity to the abovementioned MW transmission lines dedicated to driving the ESR, NMR, and EDSR transitions. A single-electron transistor (SET) is used to read out the state of the electron spin via energy-dependent tunneling (*21*), whereby the physical observable is the *z* projection of the electron spin. During the readout phase, a ∣↑〉 electron is signaled by a “blip” in current through the SET. The electron readout by spin-dependent tunneling automatically resets the electron to the ∣↓〉 state. The nuclear spin state can be inferred by measuring the electron spin after applying a frequency-selective rotation of the electron spin, conditional on the nuclear spin state. The nuclear spin-dependent electron rotation is most conveniently achieved by applying an adiabatic frequency sweep across one of the electron resonances (*22*). Further details on operation and readout of the qubit are given in Materials and Methods.

## RESULTS

### Magnetic and electric resonance spectra

**Fig. 2.**
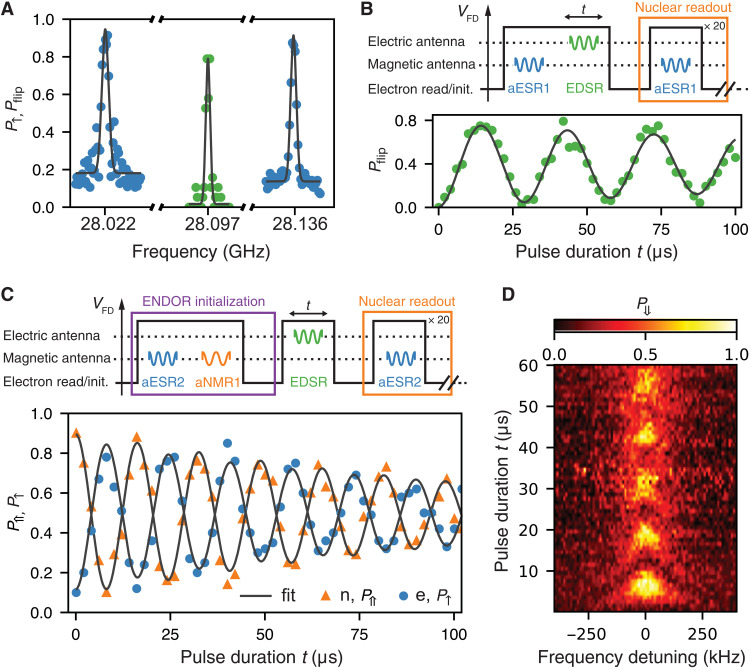
Coherent electrical drive. (**A**) The frequency spectrum shows two ESR peaks separated by *A* = 114.1 MHz. The flip-flop resonance peak is at *f*_EDSR_ = 28.0966 GHz. (**B**) Coherent EDSR Rabi oscillation obtained by reading out the nuclear spin flip probability *P*_flip_. A schematic of the pulse sequence is shown on top. (**C**) Reading out the electron and nuclear spin-up proportions simultaneously highlights the antiparallel flip-flop Rabi oscillations. A schematic of the pulse sequence is shown on top. (**D**) The Rabi chevron is mapped out by detuning the drive frequency around the resonance.

The ESR spectrum (Fig. 2A, blue dots) is obtained by initializing the electron in the ∣↓〉 state, applying a short burst of MWs to the magnetic antenna, reading the electron state, and repeating the cycle 20 times to extract the electron spin-up probability *P*_↑_ (see Materials and Methods). Repeating the above for varying frequencies of the MW drive, we find the two frequencies ν_e1_ and ν_e2_ at which the electron spin is resonantly excited to the ∣↑〉 state. Each resonance corresponds to one of the possible nuclear states, and their frequency separation directly yields the value of the electron-nuclear hyperfine coupling *A* = 114.1 MHz. This differs slightly from the value *A* = 117.53 MHz observed in bulk experiments (*23*), due to the presence of strain and strong static electric fields in our nanoelectronic device (*12*).

The flip-flop resonance is found by preparing the electron in ∣↓〉, applying an MW burst to the FD gate (Fig. 1C, left, brown), i.e., the high-frequency transmission line designed to deliver an electric field and drive EDSR transitions, and measuring the nuclear spin state. Here, the nuclear state is measured by applying an adiabatic frequency sweep (*22*) of the MW drive on the magnetic antenna around the ESR1 resonance (adiabatic transitions are labeled with the prefix “a,” e.g., aESR1 in this case), followed by electron spin readout. The ESR1 resonance is conditional on the nuclear spin being ∣⇓〉. Therefore, the electron readout after the conditional electron spin rotation represents a projective readout of the nuclear state: observing an electron ∣↑〉 projects the nucleus on ∣⇓〉, whereas observing ∣↓〉 projects the nucleus to ∣⇑〉 (*18*). We repeat the above cycle 20 times and extract the probability *P*_flip_ that the nucleus is flipped as a result of the EDSR drive, as a function of its frequency. A high probability *P*_flip_ of the nuclear state changing from one shot to the next indicates that the flip-flop resonance is being efficiently driven. Note, however, that once the system is excited to the ∣↑⇓〉 state, the electron readout via spin-dependent tunneling results in the ∣↑〉 electron being replaced by a ∣↓〉 one, which brings the electron-nuclear system to the ∣↓⇓〉 state, outside of the flip-flop qubit subspace. To prevent this, before each EDSR shot, we apply an aESR1 pulse, which returns the system to ∣↑⇓〉 if it is in the ∣↓⇓〉 state (see Materials and Methods) and leaves it unchanged (in the ∣↓⇑〉 state) otherwise. We find a high nuclear spin flip probability at *f*_EDSR_ = 28.0966 GHz (Fig. 2A), in agreement with the EDSR (flip-flop) transition frequency predicted from the values of *A*, γ_e_, and B_0_, measured independently.

### Coherent control of the flip-flop qubit

To demonstrate coherent electrical control of the flip-flop transition, we first perform an EDSR Rabi experiment, consisting of the pulse sequence shown in Fig. 2B. As before, an aESR1 prepulse is used to ensure that the electron-nuclear system remains within the flip-flop subspace after every shot. Clear Rabi oscillations are observed by plotting the nuclear flip probability *P*_flip_ as a function of the duration of the electrical EDSR pulse (Fig. 2B). The nuclear spin readout is repeated 20 times for every pulse duration, and the electron spin is left in the ∣↓〉 state after each shot.

Because the electron spin is itself read out as part of the nuclear readout process, we have the additional possibility of measuring the state of both spins and verifying the electron-nuclear flip-flop dynamics. Unlike the previous experiments, where we only measured the probability for the nucleus to flip without requiring it to start in a specific orientation, we now need to start each sequence with the deterministic initialization of the flip-flop ground state ∣↓⇑〉. We achieve this by an electron-nuclear double-resonance (ENDOR) sequence comprising an aESR2 pulse, followed by an aNMR1 pulse and a subsequent electron readout (see Figs. 1A and 2C and Materials and Methods for more details).

Once initialized, we apply a resonant EDSR tone, which drives the transition from the ∣↓⇑〉 to the ∣↑⇓〉 state, and first read out the electron spin (*21*). Subsequently, we reload an electron ∣↓〉 onto the donor and perform the nuclear spin readout (*18*) as described earlier (see Fig. 1A and pulse sequence in Fig. 2C). The sequential readout of the electron and the nuclear states is made possible by the quantum nondemolition (QND) nature of the nuclear spin readout (*18*). By repeating this sequence more than 20 times, we determine the electron *P*(↑) and nuclear *P*(⇑) spin-up proportions for each EDSR pulse duration. Figure 2C shows the antiparallel coherent drive of both electron and nuclear spins.

By detuning the drive frequency of the EDSR pulse around the resonance, we map out the Rabi chevron pattern of the nuclear spin (see Fig. 2D). Here, we again initialize the system in the flip-flop ground state ∣↓⇑〉 using the ENDOR pulse sequence.

### Coherence times and gate fidelities

**Fig. 3.**
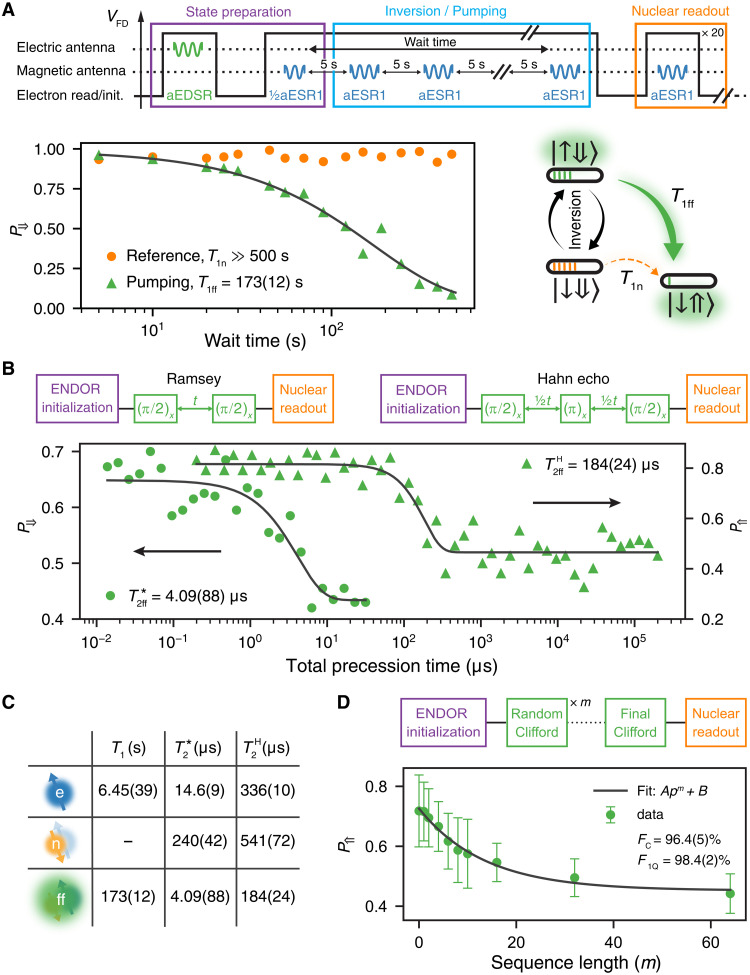
Relaxation, coherence, and gate fidelity. (**A**) The flip-flop qubit relaxation time *T*_1ff_ = 173(12) s is measured by initializing the donor in the *a*∣↑⇓〉 + *b*∣↓⇓〉 state with ∣*a*∣^2^ ≈ ∣*b*∣^2^ ≈ 0.5 and replenishing the ∣↑⇓〉 state population with adiabatic aESR1 inversion pulses applied every 5 s to counteract the electron relaxation channel ∣↑⇓〉 → ∣↓⇓〉. (**B**) Fitting the Ramsey and the Hahn echo decays using an exponential decay reveals $T_{2\mathrm{ff}}^{*}=4.09(88)$ μs (with exponent 1.28) and $T_{2\mathrm{ff}}^{H}=184(24)$ μs (exponent 2), respectively. (**C**) Tabulated values of the relaxation and coherence times measured on the electron, nuclear, and flip-flop qubits. (**D**) Randomized benchmarking (RB) experiment for the flip-flop qubit, yielding an average one-qubit gate fidelity *ℱ*_1*Q*_ = 98.4(2)%.

Having demonstrated the coherent operation and readout of the flip-flop qubit, we proceed to measure its key performance metrics for quantum information processing, i.e., relaxation, coherence, and gate fidelities. From bulk experiments on ^31^P donors, the relaxation process within the flip-flop qubit subspace (∣↑⇓〉 → ∣↓⇑〉) is known to be extremely slow, *T*_1ff_ ≈ 5 hours (*23*), but it is not obvious that it would remain unchanged in a nanoscale device subjected to strong electric fields. Because the electron spin relaxation time (∣↑⇓〉 → ∣↓⇓〉) is *T*_1e_ = 6.45(39) s in this device (here and in the rest of the manuscript, uncertainties are given as SDs), measuring *T*_1ff_ requires saturating the ESR1 transition while monitoring the decay of the system from the ∣⇓〉 subspace via the flip-flop transition. We adopted the pumping scheme depicted in Fig. 3A: Starting from the ∣↓⇓〉 state, the donor is placed in a superposition state *a*∣↓⇓〉 + *b*∣↑⇓〉, with ∣*a*∣^2^ ≈ ∣*b*∣^2^ ≈ 0.5, using a slow frequency sweep (labeled ^1^/_2_aESR1) calibrated to yield a 50% probability of inverting the electron spin (see section S1 for details) (*22*). After this, we apply aESR1 inversion pulses every 5 s to counteract the *T*_1e_ process and measure the nuclear state at the end. The probability of finding the nucleus in the initially prepared ∣⇓〉 state slowly decays with time as a consequence of the ∣↑⇓〉 → ∣↓⇑〉 flip-flop relaxation process. Fitting the decay, we obtain *T*_1ff_ = 173(12) s, much longer than *T*_1e_. We also independently verified that, without repopulating the ↑⇓ state, the rate of nuclear spin flip ∣↓⇓〉 → ∣↓⇑〉 is immeasurably slow, *T*_1n_ ≫ 500 s.

To investigate the coherence of the flip-flop qubit, we performed an on-resonance Ramsey experiment, where we initialized the system in the flip-flop ground state ∣↓⇑〉 using the ENDOR sequence and apply two consecutive EDSR π/2-pulses separated by a varying time delay (see Fig. 3B). We obtained a pure dephasing time $T_{2\mathrm{ff}}^{*}=4.09(88)$ μs, which is nearly a factor of 4 lower than $T_{2e}^{*}=14.6(9)$ μs for the donor-bound electron (Fig. 3C).

A Hahn echo experiment is performed by applying a π-pulse halfway through the free evolution time, which decouples the qubit from slow noise and extends the coherence time to $T_{2\mathrm{ff}}^{H}=184(24)$ μs (Fig. 3B). This value is approximately half the echo decay time that we measured on the electron, $T_{2e}^{H}=336(10)$ μs (Fig. 3C).

The microscopic origin of the flip-flop decoherence mechanisms, and their relation to the electron spin decoherence, is still under investigation. A key observation is that the EDSR pulses induce a transient shift of the resonance frequencies of up to 80 kHz, chiefly by affecting the electron gyromagnetic ratio (see section S2 for details). The pulse-induced resonance shift (PIRS) depends on the power and duration of the pulse in a nontrivial way and decays slowly after the pulse is turned off. Similar effects were reported earlier in the literature (*24*–*27*) and attributed to heating, rectification effects, or excitation of charge traps, but remain poorly understood.

Another key decoherence mechanism is the presence of residual ^29^Si nuclear spins in the substrate. Despite using an isotopically enriched ^28^Si material with 730 parts per million (ppm) residual ^29^Si concentration, we found the flip-flop and ESR resonances to be split into six well-resolved clusters of frequencies, indicating at least three ^29^Si nuclei coupled to the electron by ∼100 kHz (see section S3 for details). These nuclei flip as often as once per minute.

We measured the average one-qubit gate fidelities of the flip-flop qubit using the well-established methods of gate set tomography (GST) (*28*) and randomized benchmarking (RB) (*29*). In both cases, the effect of ^29^Si nuclear spin flips is mitigated by sandwiching each gate sequence between spectrum scans to monitor the instantaneous resonance frequency and only accept the measurement if the frequency remains constant (see section S4 for details). Despite this precaution, the GST analysis reveals a strong deviation from a Markovian model, possibly due to the effect of PIRS. Therefore, the GST one-qubit average fidelities ℱ_1Q_ = 97.5 to 98.5% are additionally verified by RB.

RB determines the average gate fidelity by applying to the qubit a random sequence of Clifford gates with varying length *m*. The sequence always starts by initializing the qubit in the ∣↓⇑〉 state, using the ENDOR sequence described before. In our compilation, the Clifford gates are composed of ≈2.233 native gates ∈{*X*, *Y*, ± *X*_π/2_, ± *Y*_π/2_} on average. The last Clifford operation in each sequence is chosen such that the final state ideally returns to ∣↓⇑〉. The final state of the flip-flop qubit is measured by monitoring the probability *P*_⇑_ of finding the nuclear spin in the ∣⇑〉 state. In the presence of gate errors, *P*_⇑_ decays as a function of sequence length *m*, and the average gate fidelity is determined from the decay rate (see Fig. 3C and section S4 for further details). We find an average Clifford gate fidelity ℱ_C_ = 96.4(5)%, which corresponds to an average native gate fidelity ℱ_1Q_ = 98.4(2)% (Fig. 3D). Comparing gate times (1 to 6 μs) to the coherence time of $T_{2\mathrm{ff}}^{*}=4.09(88)$ μs, we conclude that the gate fidelities are limited by decoherence effects, which is further confirmed by GST (see section S4 for details).

### Calibration and verification of the electrical drive

**Fig. 4.**
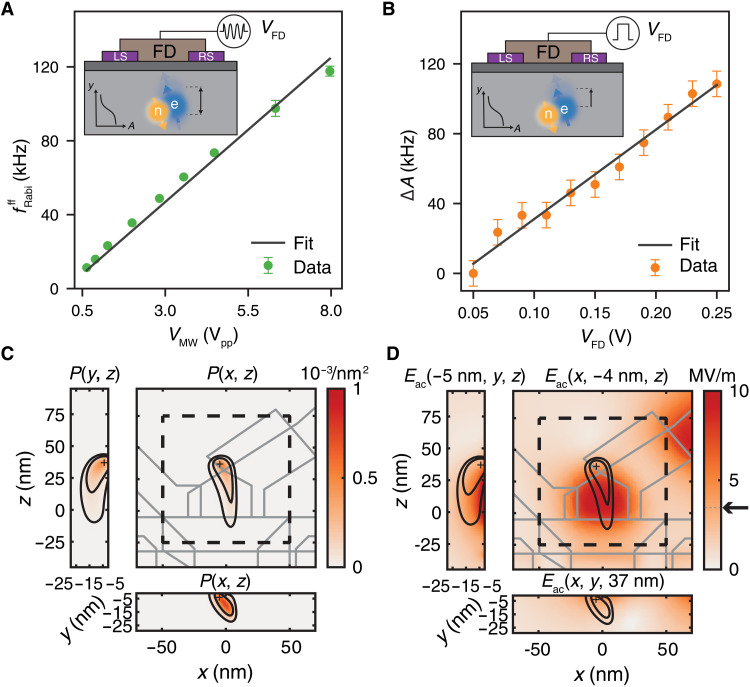
Electrical drive via hyperfine modulation. (**A**) Linear dependence of the flip-flop Rabi frequencies on the voltage at the output of the MW source. (**B**) Stark shift of the hyperfine coupling produced by a dc voltage applied to the FD gate, as extracted from the shift of the NMR1 resonance frequency. An independent calibration of the line attenuation at MW confirms that the flip-flop qubit is driven by dynamic modulation of the hyperfine coupling. (**C**) Triangulation of the most probable location of the donor under study, obtained through COMSOL finite-element models informed by the capacitive coupling between the donor and each electrostatic gate (see section S5). The contours indicate the 1σ and 2σ confidence regions. (**D**) Amplitude of the MW electric field *E*_ac_ around the donor location, estimated using the same COMSOL model as above, assuming a voltage on the FD gate *V*_FD_ = 1 V_pp_. We find *E*_ac_ ≈ 3.5 MV/m at the most likely donor location.

We proceed to investigate the physical origin of the electric drive of the flip-flop qubit by comparing Rabi frequencies $f_{\mathrm{Rabi}}^{\mathrm{ff}}$ to the hyperfine Stark shift ∂*A*(*E*)/*∂E*. If the driving mechanism is the electrical modulation of the hyperfine coupling, the two should be linked by the simple relation $f_{\mathrm{Rabi}}^{\mathrm{ff}}=[\partial A(E)/2\partial E]E_{\mathrm{ac}}$. Figure 4A shows a linear dependence of the flip-flop Rabi frequency on the peak-to-peak voltage applied to the electrical antenna, indicating that we are within the rotating wave approximation. For the highest MW driving power (22 dBm at the source, corresponding to 8 V_pp_ (peak-to-peak voltage) amplitude), we reach a flip-flop Rabi frequency $f_{\mathrm{Rabi}}^{\mathrm{ff}}=118.5(25)$ kHz, which is a factor of 5 higher than the fastest single-nucleus Rabi frequency reported in the literature (*18*).

It is in general difficult to estimate the precise value of the oscillating voltage at the tip of the electrical antenna, due to the strongly frequency-dependent losses of the transmission line between the MW source and the device. Here, however, we can correlate $f_{\mathrm{Rabi}}^{\mathrm{ff}}$ with the dc Stark shift of the hyperfine coupling, which can be measured independently. We apply a dc voltage shift Δ*V*_FD_ to the FD gate (the same used for EDSR) and measure the hyperfine Stark shift Δ*A*(Δ*V*_FD_) through the shift of the NMR1 resonance, *f*_NMR1_ = *A*/2 + γ_n_B_0_ (Fig. 4B). A linear fit to the data yields *∂A*/*∂V*_FD_ = 512(26) kHz/V. We have performed a capacitance-based triangulation of the donor position (see section S5 for details) and found that it is located next to one of the side confining gates (Fig. 4C). Therefore, a positive voltage on the tip of the FD gate may bring the electron laterally closer to the nucleus (*15*) and lead to the positive slope, instead of pulling the electron away from the donor nucleus (*13*) and reducing *A*. Using the experimentally determined slopes $\partial f_{\mathrm{Rabi}}^{\mathrm{ff}}/\partial V_{\mathrm{FD}}$ and ∂Δ*A*/*∂V*_FD_ from Fig. 4 (A and B) and the formula for the EDSR Rabi frequency, we determine that the total line attenuation between MW source and FD gate at 28 GHz is 18.1(5) dB. This value is in good agreement with an independent estimate, 19.4(5) dB, obtained by comparing the effect of a 100-Hz square pulse and the 28-GHz MW pulse on the broadening of the SET Coulomb peaks (see section S6 for details). The slight discrepancy can be explained by the different capacitive couplings of the FD gate to the donor-bound electron and to the SET. This experiment thus confirms that the flip-flop qubit is driven by the electrical modulation of the hyperfine coupling.

The triangulation of the donor location further allows for an estimation of the peak electric fields at the donor site during the electric drive (see Fig. 4D). We find that applying *V*_MW_ = 8 V_pp_ to the FD gate (which yields $f_{\mathrm{Rabi}}^{\mathrm{ff}}=118$ kHz) produces oscillating electric fields *E*_ac_ of order 5 MV/m in the active region of the device, with *E*_ac_ ≈ 3.5 MV/m at the most likely donor location. This means that the flip-flop transition in the donor used in this experiment can be electrically driven at a rate of ≈30 kHz for an *E*_ac_ = 1 MV/m oscillating electric field.

### Extension to spin ensembles: Nuclear hyperpolarization

Accessing the flip-flop transition opens interesting possibilities for experiments on ensembles of coupled electron-nuclear spin systems. It is generally very challenging to directly detect the induction signal from very dilute nuclear spins, like in weakly doped Si crystals, unless one uses nuclear hyperpolarization. The hyperpolarization of ensembles of ^31^P nuclear spins has been achieved by magnetically (*30*) or optically exciting the donor-bound electrons (*31*–*33*), to the point where direct inductive detection of the NMR signal becomes possible (*34*). However, the microscopic processes that underpin such phenomena are complicated and often slow, unless other fast-relaxing electron spins are involved (*35*).

Our experiments on a single ^31^P atom allow us to predict that the flip-flop transition could be used to efficiently hyperpolarize spin ensembles, via a scheme similar to the “solid effect” (*30*) but where the ∣↓⇑〉 → ∣↑⇓〉 transition is not forbidden when driven electrically. A high population of the ∣↓⇓〉 state can be obtained by first transferring the population of ∣↓⇑〉 to ∣↑⇓〉 via an adiabatic EDSR flip-flop transition and then relaxing the electron spin from ∣↑⇓〉 to ∣↓⇓〉 through above-bandgap optical excitation, which quickly resets the electron polarization to its thermal equilibrium (*36*, *37*).

Bulk donors only have a quadratic Stark shift of the hyperfine coupling, *A*(*E*) = η*E*^2^ (*20*). This hyperpolarization method would thus require driving the EDSR transition at half the flip-flop resonance frequency (*38*), posing technical problems if one wished to further manipulate the electron spin at the same time. To circumvent the issue, we have designed a rectangular three-dimensional (3D) cavity that is capable of delivering strong electric and magnetic fields while allowing for an electrical dc bias to be applied to the sample (see section S7 for details). A suspended copper block concentrates the TE_101_ cavity mode to the space between the block and cavity walls. The sample is mounted in a region with both strong electric and magnetic fields, *E*_ac_≈ 0.2 MV/m and *B*_ac_≈ 1 mT at 50 W input power, respectively. By dc biasing the copper block, one can induce the linear hyperfine Stark effect that enables EDSR drive directly at the flip-flop resonance frequency, as in the present single-atom experiment. The predicted value of *E*_ac_ in the cavity, combined with the experimental values of $f_{\mathrm{Rabi}}^{\mathrm{ff}}$, would allow driving the ∣↓⇑〉 → ∣↑⇓〉 transition in submillisecond times. A resonator bandwidth of ≈60 MHz at a magnetic field of 0.35 T (MW X-band, *f* ≈ 10 GHz), i.e., a quality factor *Q* ≈ 150, would allow simultaneously addressing the flip-flop transition for dynamic nuclear polarization and the ESR transition for readout to verify the hyperpolarization.

## DISCUSSION

Future experiments will focus on operating the flip-flop qubit in the regime of large electric dipole, where the wave function of the electron is equally shared between the donor and an interface quantum dot (*13*). This regime is predicted to yield fast one-qubit (30 ns for a π/2 rotation) and two-qubit (40 ns for a $\sqrt{i\mathrm{SWAP}}$) operations with fidelities well above 99% under realistic noise conditions, with further improvements possible using optimal control schemes (*39*). The present setup did not allow reaching the large-dipole regime because of the presence of many other donors randomly implanted in the device: The large gate voltage swing necessary to move the electron away from the donor under study would unsettle the charge state of nearby donors (see section S8 for details). The recent demonstration of deterministic single-ion implantation with 99.85% confidence (*40*) will eliminate this problem in future devices.

In the present experiment, an on-chip antenna to deliver oscillating magnetic fields remains necessary to perform NMR control and ensure that the system is prepared in the flip-flop subspace. In the future, replacing the ^31^P donor with one of the heavier group V donors (^75^As, ^121^Sb, ^123^Sb, or ^209^Bi) would provide a nuclear spin with *I* > 1/2. The presence of an electric quadrupole moment in *I* > 1/2 nuclei enables direct control of the nuclear spin through the recently demonstrated method of nuclear electric resonance (NER) (*12*). The flip-flop transition presented here adds another mechanism for electrical control of donor spins. The combination of electron-nuclear flip-flop transitions and NER will permit the control of the whole Hilbert space of all group V donors other than ^31^P using solely electric fields. The flip-flop drive can also be used to implement geometric two-qubit logic gates for nuclear spins, which have recently shown to yield universal quantum logic with fidelities above 99% (*41*).

The results shown here already illustrate the broad applicability of the flip-flop qubit idea, even to atoms and atom-like systems that do not permit the creation of a large electric dipole or do not have anisotropic hyperfine couplings. For example, all-epitaxial donor devices fabricated with scanning probe lithography (*42*) do not allow the formation of interface quantum dots, but their flip-flop states could be electrically controlled using the methods shown here. Electrical control of the flip-flop transition has already been used to hyperpolarize the nuclear spins of individual Cu atoms on a surface using a scanning tunneling microscope (*43*) and may be extended to hyperpolarize ensembles of nuclear spins using a specially designed 3D cavity as discussed here. Atoms (*44*) and atom-like defects (*45*) in SiC have strong electrical tunability of their electronic states, which may be exploited for flip-flop transitions in the presence of hyperfine-coupled nuclei. Molecular systems could permit even more tailored electrical responses (*46*).

## MATERIALS AND METHODS

### Device fabrication

**Fig. 5.**
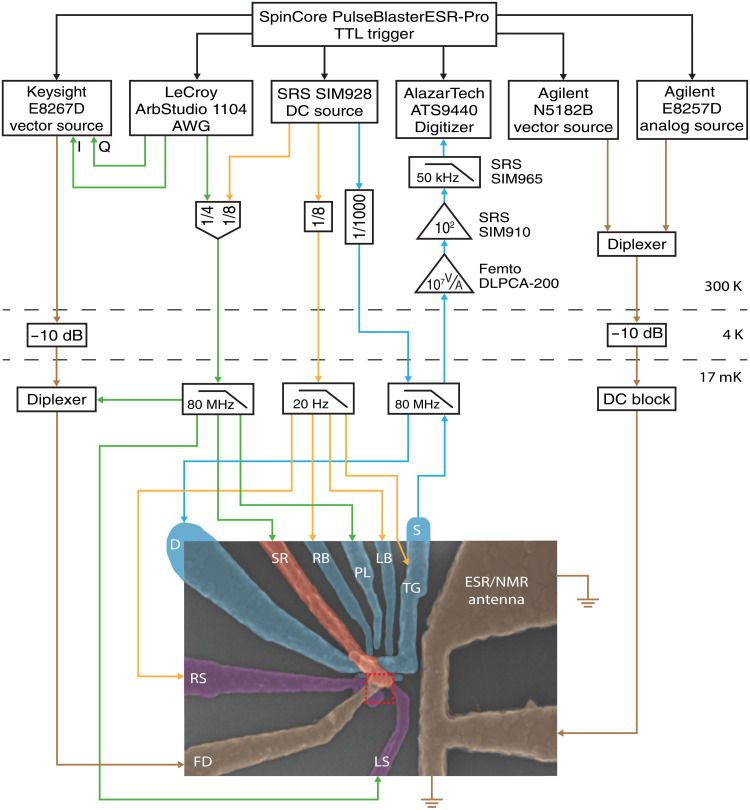
Experimental setup. Wiring and instrumentation used to control and read out the donor spin qubit. The red dashed square defines the implantation region for this qubit device.

The device under investigation is fabricated on an isotopically enriched ^28^Si wafer. We fabricate metal-oxide nanostructures in the proximity to the implantation area of the ^31^P donors (Fig. 5, red dashed square) to manipulate and read out the donor spin qubit, similar to (*17*, *18*, *21*). A SET (Fig. 5, cyan) is used to read out the spin states of the donor-bound electron (*21*) or the nucleus (*18*). The device structure also comprises a broadband on-chip MW magnetic antenna (Fig. 5, right, brown) to drive the spins via standard nuclear NMR and ESR techniques. Local gates are added to tune the tunnel coupling between the donor and the SET [“SET rate” gate (SR), red] and the coupling between the donor and interface quantum dot by laterally shifting the electron wave function [“right side” (RS) and “left side” (LS) gates, purple] (*13*). The “FD” gate overlapping the implantation area is used as an electrical antenna (Fig. 5, left, brown) to apply an electrical MW drive tone.

The fabrication procedure of the donor qubit device follows the recipe outlined in (*17*, *41*). Here, we provide a short summary containing important implantation parameters and dimensions of the nanostructures. The natural silicon wafer that is used in this work contains a 900-nm-thick isotopically enriched ^28^Si epitaxial layer (residual ^29^Si concentration of 730 ppm) on top of a lightly p-doped natural Si handle wafer. Using optical lithography and thermal diffusion of phosphorus (boron), n^+^ (p) regions are defined on the sample. The n^+^-type regions serve as electron reservoirs for the qubit device and are connected to aluminum ohmic contacts. The p-doped regions are added to prevent leakage between ohmic contacts. Using wet thermal oxidation, a 200-nm SiO_2_ field oxide is grown on top of the substrate. The active device region is defined by etching a 30 μm–by–60 μm area in the center of the field oxide using HF (hydrofluoric) acid that is subsequently covered by an 8-nm high-quality SiO_2_ gate oxide in a dry thermal oxidation step. We define a 100 nm–by–90 nm window (Fig. 5, red dashed square) in a 200-nm-thick poly(methyl methacrylate) resist using electron-beam lithography (EBL), through which the ^31^P^+^ ions are implanted at a 7° angle from the substrate norm. For this sample, we use an implantation energy of 10 keV and a fluence of 1.4 × 10^12^ atoms/cm^2^. According to Monte Carlo SRIM (Stopping and Range of Ions in Matter) (*47*) simulations, approximately 40 donors are implanted within the window region ≈ 3.5 to 10.1 nm deep below the SiO_2_/Si interface. To activate the donors and repair the implantation damage, we use a rapid thermal anneal at 1000°C for 5 s. To avoid potential leakage through the thin SiO_2_ layer, we deposit an additional 3-nm Al_2_O_3_ layer via atomic layer deposition. The qubit device itself is defined in four EBL steps each including a thermal deposition of aluminum gates (with increasing thickness of 25, 25, 50, and 80 nm). After each deposition, the sample is exposed to a pure 100-mtorr oxygen gas for 3 min to oxidize an insulating Al_2_O_3_ layer between the gates. We connect all gate electrodes from all layers electrically to avoid electrostatic discharge damage (the shorts are broken after wire bonding using a diamond scriber). Last, we anneal the qubit devices at 400°C in a forming gas (95% N_2_/5% H_2_) atmosphere for 15 min to passivate interface traps and repair EBL damage.

### Measurement setup

The sample is wire-bonded to a gold-plated printed circuit board inside a copper enclosure. The enclosure is mounted onto a cold finger attached to the mixing chamber of a Bluefors BF-LD400 dilution refrigerator and is cooled down to ≈17 mK. The sample is placed in the center of a superconducting magnet, operated in persistent mode at a magnetic field between B_0_≈ 0.9 to 1 T. The field is applied along the short-circuit termination of the magnetic (ESR and NMR) antenna, parallel to the surface of the chip and to the [110] Si crystal direction. A schematic of the experimental setup is shown in Fig. 5. Static dc voltages from battery-powered Stanford Research Systems (SRS) SIM928 voltage sources are used to bias the metallic gate electrodes via homemade resistive voltage dividers at room temperature. The SET top gate (TG), left barrier (LB), right barrier (RB), and right side (RS) gates are connected via second-order low-pass RC filters with a 20-Hz cutoff. Gates used for loading/unloading the donor, i.e., the plunger (PL), LS, SR, and FD gates, are filtered by a seventh-order low-pass LC (inductance-capacitance) filter with a 80-MHz cutoff frequency. All filters are thermally anchored at the mixing chamber stage of the dilution refrigerator. Baseband pulses from a LeCroy ArbStudio 1104 arbitrary waveform generator (AWG) are added via room temperature resistive voltage combiners to FD and SR. The ESR MW signals are generated by an Agilent E8257D 50 GHz analog source, and we use an Agilent N5182B 6-GHz vector source to create rf signals for NMR control. Using a Marki Microwave DPX-1721 diplexer at room temperature, both high-frequency signals are routed to the magnetic antenna via a semirigid coaxial cable. A 10-dB attenuator is used for thermal anchoring at the 4 K stage.

The MW signal for EDSR control is generated by a Keysight E8267D 44-GHz vector source. For single-sideband IQ modulation, rf pulses from the LeCroy ArbStudio 1104 AWG are fed to the in-phase (I) and quadrature-phase (Q) ports of the vector source. The high-frequency signal is attenuated by 10 dB at the 4 K stage and combined to the baseband control pulses at the mixing chamber using a Marki Microwave DPX-1721 diplexer. The combined signal is routed to the FD gate (electric/EDSR antenna).

The SET current is amplified using a room temperature Femto DLPCA-200 transimpedance amplifier (10^7^ VA^−1^ gain, 50-kHz bandwidth) and an SRS SIM910 JFET amplifier (100 VV^−1^ gain). The amplified signal is filtered using an SRS SIM965 analog 50-kHz low-pass Bessel filter and digitized by an AlazarTech ATS9440 PCI card. The above instruments are triggered by a SpinCore PulseBlasterESR-Pro. Software control of the measurement hardware and the generation of pulse sequences is done in Python using the QCoDeS (*48*) and SilQ (*49*) framework.

### Electron spin readout and initialization

The spin of the donor-bound electron is read out using energy-dependent tunneling into a cold electron reservoir. Because of the large electron Zeeman splitting in our experiment, this translates into a measurement of the *S_z_* spin eigenstates, ∣↓〉 and ∣↑〉. The method is a modified version of the well-known Elzerman readout scheme (*50*, *51*). The modification consists of using the island of the SET charge sensor as the cold charge reservoir that discriminates the spin eigenstates (*21*, *51*), rather than having separate charge sensors and charge reservoir.

**Fig. 6.**
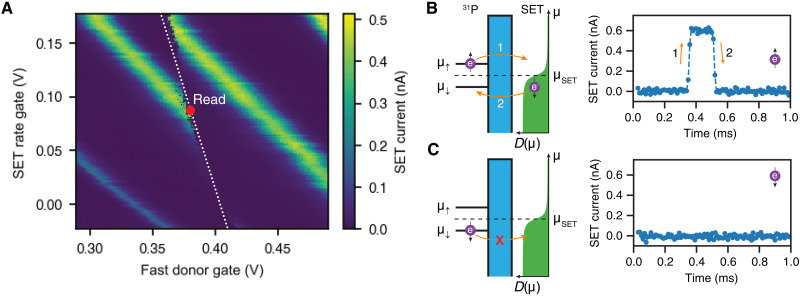
Electron spin-dependent tunneling. (**A**) SET current as a function of two gate voltages. The pattern of Coulomb peaks (green) is broken (white dotted line) in the presence of a donor charge transition. The read position for the electron spin is indicated by a red dot and corresponds to the point where the donor electrochemical potential is equal to that of the SET island. (**B** and **C**) Schematic depiction of the electron spin-dependent tunneling between the donor and the SET island (left) with the corresponding SET current traces (right). The ^31^P donor is tunnel-coupled to the SET island with a potential barrier between them shown in cyan. The Fermi-Dirac distribution for the density of the occupied states at the SET is shown in green.

The white dotted line in Fig. 6A highlights a donor charge transition. To perform electron spin readout and initialization, we tune the system into the so-called “read” spot (Fig. 6A, red dot), where the electrochemical potentials of the SET island μ_SET_ and the ^31^P donor μ_P_ = (μ_↑_ + μ_↓_)/2 are aligned, i.e., μ_SET_ = μ_P_.

In a static magnetic field **B**_0_ (≈1 T), the Zeeman interaction splits the electrochemical potential of the donor into two energy levels, μ_↓_ and μ_↑_, for the two electron spin ∣↓〉 and ∣↑〉 states, respectively. In this case, μ_↑_ > μ_SET_ > μ_↓_, and only the electron in the ∣↑〉 state is energetically allowed to tunnel from the donor to the SET, as there are no available states below μ_SET_ at the SET island (see Fig. 6B). During this tunnel event (Fig. 6B, left, 1), the Coulomb blockade regime is lifted, and we detect an increase in the SET current (Fig. 6B, right). Because μ_↓_ is the only level at the donor below μ_SET_, only an electron in the ∣↓〉 state can tunnel back from the SET to the donor (Fig. 6B, left, 2). In this case, the SET returns to the blockade regime and *I*_SET_ = 0 again (Fig. 6B, right). The obtained current spike, which we call a “blip,” is then used as a spin-readout signal. It tells us that the electron at the donor was in the ∣↑〉 state and is now initialized in the ∣↓〉 state. If we do not detect any blips, it means the electron at the donor is in the ∣↓〉 state and does not tunnel anywhere (Fig. 6C). Therefore, this spin-dependent tunneling of the electron between the donor and the SET provides a single-shot readout and initialization of the electron spin into the ∣↓〉 state (*21*).

The electron spin-up proportion *P*(↑) is then calculated by averaging over multiple repetitions. Further details of donor electron spin readout and initialization can be found in (*21*, *51*, *52*).

### Nuclear spin readout

The donor-bound electron can be used as an ancilla qubit to read out the state of the nuclear spin via QND measurement with fidelities exceeding 99.99% (see later section for more details).

The hyperfine interaction between the electron and the nucleus results in two electron resonance frequencies $f_{\mathrm{ESR}1}=\gamma_{e}B_{0}-\frac{A}{2}\;\mathrm{and}\;f_{\mathrm{ESR}2}=\gamma_{e}B_{0}+\frac{A}{2}$, depending on the nuclear spin being ∣⇓〉 or ∣⇑〉 (*18*). Starting from a ∣↓〉 electron, an adiabatic ESR inversion pulse (*22*) at either ESR frequency results in a ∣↑〉 electron if the nuclear spin was in the state corresponding to ESR frequency being probed. In other words, the ESR inversion constitutes a controlled-X logic operation on the electron, conditional on the state of the nucleus. We perform the electron inversion using an adiabatic frequency sweep across the resonance (*22*) to be insensitive to small changes in the instantaneous resonance frequency.

Reading out the electron spin after an inversion pulse determines the nuclear state in a single shot (we call this readout a “shot”). The fidelity of this readout process is the product of the single-shot electron readout fidelity and the fidelity of inverting the electron via an adiabatic pulse.

Because γ_e_B_0_ ≫ *A*, the electron-nuclear hyperfine coupling is well approximated by *AS_z_I_z_*, meaning that the interaction commutes with the Hamiltonian of the nuclear spin. This is the quintessential requirement of a QND measurement (*18*, *53*). In practice, it means that the nuclear spin will be found again in the same eigenstate after the first shot. We can thus repeat it multiple times, i.e., perform multiple measurement shots, to improve the nuclear readout fidelity. We calculate the electron spin-up proportion over all shots and determine the nuclear state by comparing the spin-up proportion to a threshold value (typically around 0.4 to 0.5).

For nuclear spin qubit manipulations that do not depend on the initial state (e.g., Rabi drive), measuring the nuclear spin flip probability instead of the actual spin state is sufficient. For this, we read out the nuclear state *N*_samples_ times (typically *N*_samples_ ≥ 20) and calculate how many times the nuclear spin flipped (*N*_flip_) in two consecutive measurements. The flip probability is then determined as *P*_flip_ = *N*_flip_/(*N*_samples_ − 1).

### Nuclear spin initialization

**Fig. 7.**
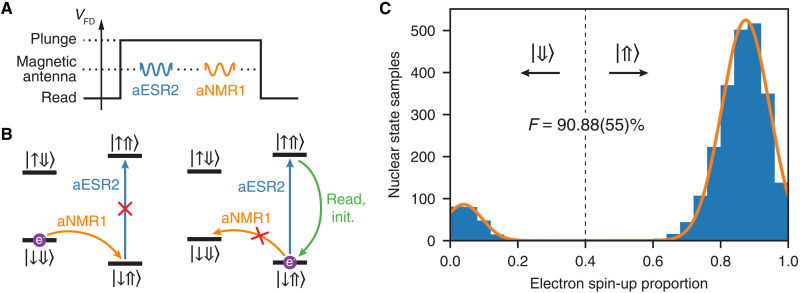
Nuclear spin initialization fidelity. ∣⇑〉 (**A**) ENDOR sequence containing consecutive aESR2 and aNMR1 pulses. The sequence starts and ends in the read phase to initialize the electron into the ∣↓〉 state. (**B**) Energy level diagrams for the ^31^P donor show nuclear spin initialization into the ∣⇑〉 state when we apply the ENDOR sequence in (A). With the electron initialized in the ∣↓〉 state in the read phase, we consider two cases of initial nuclear states before the ENDOR sequence: ∣⇓〉 (left diagram) and ∣⇑〉 (right diagram). (**C**) The histogram shows the final nuclear state after an ENDOR initialization sequence followed by nuclear spin readout with 25 shots. The nuclear spin is randomized after the 25 shots to test the ENDOR sequence again. We assign to ∣⇑〉 the instances where the probability *P*_↑_ of finding the electron in the ∣↑〉 state is above 0.4. Samples where *P*_↑_ < 0.4 threshold correspond to the nuclear spin ∣⇓〉 state, i.e., to the error of the ENDOR initialization. The histogram yields an ENDOR initialization fidelity of *F* = 90.88(55)%. The nuclear readout fidelity can be inferred by fitting the histogram peaks and calculating their overlap, yielding *F*_read_ > 99.99%.

For benchmarking and quantum logic experiments, we need to initialize the flip-flop qubit into the ∣↓⇑〉 state. We have seen that reading out the electron state also initializes it into the ∣↓〉 state. To deterministically initialize the nucleus into the ∣⇑〉 state, and hence the flip-flop qubit into the ∣↓⇑〉, we make use of an ENDOR sequence. The ENDOR sequence comprises an aESR2 pulse, an aNMR1 pulse, and an electron readout (see Fig. 7A). We use adiabatic pulses that sweep around the actual resonance frequency and adjust the frequency range such that the pulses are insensitive to frequency deviations. On the one hand, if the system is in the ∣↓⇓〉 state, the aESR2 pulse is off-resonant and the aNMR1 pulse flips the nuclear spin to the ∣⇑〉 state (Fig. 7B, left). If, on the other hand, we already start in the ∣↓⇑〉 state, the aESR2 pulse inverts the electron spin and the aNMR1 pulse is off-resonant (Fig. 7B, right). After reading out and initializing the electron to the ∣↓〉 state, the flip-flop qubit is initialized into the ∣↓⇑〉 ground state.

To quantify the nuclear spin-up probability *P*(⇑), we perform nuclear readout and determine the nuclear spin state by comparing the electron spin-up proportion (usually ≥ 20 shots) to a threshold value. By averaging over *N*_samples_ ≥ 20 repetitions, we derive *P*(⇑).

The fidelity of the ENDOR sequence is mostly limited by the errors of electron spin initialization in the ∣↓〉 state because the fidelity of ESR/NMR inversion pulses is typically >98%. To measure these errors, we apply the ENDOR sequence and determine the nuclear state by measuring the electron spin-up proportion *P*(↑). We repeat this experiment 2500 times and apply multiple adiabatic NMR1 pulses after every 25 measurements to scramble the nuclear spin state. We determine the ENDOR fidelity to be *ℱ* = 90.88(55)% by calculating how many times we end up in the desired ∣⇑〉 state. The confidence interval shown in brackets represents the SD, which is calculated by dividing 2500 experiments into 100 independent measurements (25 experiments each) of the nuclear spin-up state probability *P*(⇑). The histogram of nuclear readouts is shown in Fig. 7C. The measured fidelity is in good agreement with the off-resonant nuclear spin-up probability of *P*(⇑) ≈ 0.1 that we see in EDSR spectrum scans.

## Supplementary Materials

### This PDF file includes:

Supplementary Text Sections S1 to S8 Figs. S1 to S13 Tables S1 to S3 References
