# Supplementary material for: An electrically driven single-atom “flip-flop” qubit
Source: Sci Adv. 2023 Feb 10;9(6):eadd9408. doi: 10.1126/sciadv.add9408 (PMC9916988; doi:10.1126/sciadv.add9408)
Supplement: Supplementary file 1 — Supplementary Text Sections S1 to S8 Figs. S1 to S13 Tables S1 to S3 References [file sciadv.add9408_sm.pdf]

Science Advances
AAAS

Supplementary Materials for

An electrically driven single-atom “flip-flop” qubit

Rostyslav Savytskyy *et al.*

Corresponding author: Andrea Morello, a.morello@unsw.edu.au

Sci. Adv. **9**, eadd9408 (2023)
DOI: 10.1126/sciadv.add9408

This PDF file includes:

Supplementary Text
Sections S1 to S8
Figs. S1 to S13
Tables S1 to S3
References

Supplementary Text

S1: Electron and flip-flop relaxation

Early experiments on ^{31}P donor ensembles in bulk silicon have shown an extremely slow relaxation process within the flip-flop qubit subspace ($|\uparrow\Downarrow\rangle \rightarrow |\downarrow\Uparrow\rangle$), $T_{1\mathrm{ff}} \approx 5$ hours [23] (note that the flip-flop process was labeled T_x in the old literature).

Here, however, we deal with a near-surface donor, in close proximity to an oxide interface and several metallic gates. In the limit where the donor-bound electron hybridizes with an interface quantum dot, the flip-flop relaxation time $T_{1\mathrm{ff}}$ can be reduced significantly [13, 54]. We thus set out to measure both the electron, $T_{1\mathrm{e}}$, and the flip-flop relaxation times directly.

We first determine the total electron spin relaxation time

$$\frac{1}{T_1} = \frac{1}{T_{1\mathrm{e}}} + \frac{1}{T_{1\mathrm{ff}}}. \tag{S.1}$$

We use a combination of aEDSR, electron read and aESR1 pulses to initialize the system in the excited $|\uparrow\Downarrow\rangle$ flip-flop state. The pulse sequence is shown in SFig. 1 A. We determine T_1 by measuring the electron decay from the $|\uparrow\Downarrow\rangle$ state which we fit to an exponential function $P\exp(-t/T_1) + P_{\mathrm{offset}}$. Here, P_{offset} is the electron spin-up proportion when the electron spin is fully decayed into the spin $|\downarrow\rangle$ state, and P is the measurement contrast between the spin $|\uparrow\rangle$ and $|\downarrow\rangle$ states. We find $T_1 = 6.45(39)$ s, which is comparable to the typical electron spin relaxation times ($T_1 \approx T_{1\mathrm{e}}$) in ^{31}P donor qubit devices [55]. According to Eq. S.1, this suggests that $T_{1\mathrm{ff}} > T_{1\mathrm{e}}$ and the flip-flop relaxation process gives a negligible contribution to the total electron spin relaxation rate.

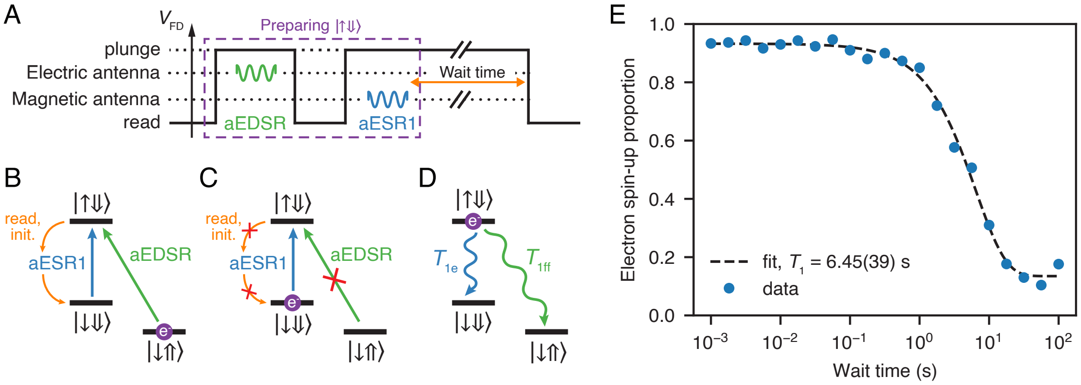

Supplementary Figure 1: **Electron spin relaxation.** (**A**) A combination of aEDSR, electron initialization and aESR1 pulses is used to prepare the excited $|\uparrow\Downarrow\rangle$ flip-flop state. (**B**) If the system is in $|\downarrow\Uparrow\rangle$, the pulse sequence prepares $|\uparrow\Downarrow\rangle$ via $|\downarrow\Uparrow\rangle \rightarrow |\uparrow\Downarrow\rangle \rightarrow |\downarrow\Downarrow\rangle \rightarrow |\uparrow\Downarrow\rangle$. (**C**) If the electron is in $|\downarrow\Downarrow\rangle$ initially, the aEDSR pulse is off-resonant and we flip the electron spin to $|\uparrow\Downarrow\rangle$ via the aESR1 pulse. (**D**) The excited flip-flop $|\uparrow\Downarrow\rangle$ state can relax via two processes: electron spin relaxation into the $|\downarrow\Downarrow\rangle$ state (blue, $T_{1\mathrm{e}}$) and hyperfine-mediated flip-flop relaxation into the $|\downarrow\Uparrow\rangle$ state (green, $T_{1\mathrm{ff}}$). (**E**) The electron spin-up proportion as a function of the wait time in (**A**) shows the electron spin decay out of the $|\uparrow\rangle$ state. By fitting an exponential function (see text) to the data, we determine the relaxation time $T_1 = 6.45(39)$ s, encompassing both the $T_{1\mathrm{e}}$ and the $T_{1\mathrm{ff}}$ processes.

Therefore, measuring $T_{1\mathrm{ff}}$ requires a method to prevent the electron spin relaxation $|\uparrow\Downarrow\rangle \rightarrow |\downarrow\Downarrow\rangle$ from bypassing the flip-flop process. We counteract the $T_{1\mathrm{e}}$ process by applying the pulse sequence shown in SFig. 2 A. We first prepare $|\downarrow\Downarrow\rangle$ using an aEDSR pulse and an electron initialization pulse. We then create a superposition state $|\psi_{\mathrm{s}}\rangle = a\,|\downarrow\Downarrow\rangle + b\,|\uparrow\Downarrow\rangle$ with equal population (i.e. ($|a|^2 \approx |b|^2 \approx 0.5$)) of the electron in the $|\uparrow\Downarrow\rangle$ and $|\downarrow\Downarrow\rangle$ states by applying a 1/2aESR1 pulse, i.e. a semi-adiabatic frequency sweep,

with rate calibrated to yield an $\approx 50\%$ probability of exciting the electron from the $|\downarrow\Downarrow\rangle$ to the $|\uparrow\Downarrow\rangle$ state. Then, by repeatedly applying full-inversion aESR1 pulses (SFig. 2 B), we periodically reverse the effect of the $|\uparrow\Downarrow\rangle \to |\downarrow\Downarrow\rangle$ relaxation channel, effectively saturating the ESR1 transition. Due to memory limitations of the AWG we are only able to apply inversion pulses every 5 s (see SFig. 2 A). Numerical simulation shows that this sequence leads to oscillations of the $|\uparrow\Downarrow\rangle$ state population between ≈ 0.23 and ≈ 0.76 with a mean value of ≈ 0.46 (see SFig. 2 D), calculated by considering a 98% fidelity of the inversion pulses and the electron spin relaxation time $T_{1\mathrm{e}} \approx 6.45$ s.

We then observe the flip-flop relaxation (SFig. 2 C), i.e. the $|\uparrow\Downarrow\rangle \to |\downarrow\Uparrow\rangle$ process, by measuring the nuclear $|\Downarrow\rangle$ state probability as a function of the wait time between the 1/2aESR1 pulse and the last aESR1 pulse in the sequence (see SFig. 2 A and E). By fitting an exponential function to the data in SFig. 2 E we find $T_{1\mathrm{ff}} = 173(12)$ s. As predicted earlier, the flip-flop relaxation time $T_{1\mathrm{ff}} \gg T_{1\mathrm{e}}$ and is indeed not a limiting factor in the flip-flop qubit operations discussed in this work. To rule out any nuclear relaxation process, we also perform a reference measurement, where we omit the inversion pulses after the 1/2aESR1 pulse. In this case, the system simply relaxes into the $|\downarrow\Downarrow\rangle$ state and we observe no further leakage out of that state, i.e. no $|\downarrow\Downarrow\rangle \to |\downarrow\Uparrow\rangle$ process, yielding $T_{1\mathrm{n}} \gg 500$ s, see SFig. 2 C, E.

S2: Pulse induced resonance shifts

In this section, we investigate the physical origin of the difference in coherence times between the donor-bound electron qubit and the flip-flop qubit. We find that applying an electric drive simultaneously with the magnetic drive decreases the electron Rabi and Hahn echo coherence times, but not the Ramsey coherence time. For strong electric drive tones, we observe a frequency shift of the ESR, NMR and EDSR resonance frequencies depending on the duration and amplitude of the electric tone. The physical origin of those pulse-induced resonance frequency shifts (PIRS) [25] is not yet understood. Below we discuss further data on the present device, which clearly unveil the presence of PIRS and highlight some of its empirical features.

Coherence measurements

The coherence times of the flip-flop qubit (summarized in Fig. 4 C) are consistently shorter than those of the electron spin qubit, including a shorter decay time of the driven Rabi oscillations, 100 μs for the flip-flop qubit compared to the ≥ 400 μs for the electron.

Shorter flip-flop coherence times are to be expected if the system is operated in the large electric dipole regime, where the electron is significantly displaced from the donor nucleus, towards the Si/SiO_2 interface. That regime increases the system exposure to charge noise, although theory models predict the existence of a second-order clock transition where coherence may be protected [13]. Here, however, we operate the donor qubit in a near-bulk regime (see S9), so one might expect the flip-flop decoherence to be dominated by the electron spin effects alone. On the other hand, the application of strong microwave electric fields may introduce effects that are not generally present when driving an electron spin qubit with oscillating magnetic fields.

To investigate these effects, we measure the electron spin Rabi, Ramsey and Hahn echo times while applying an electric ($\mathrm{EDSR}^{\mathrm{off}}$) tone simultaneously with the magnetic drive used for ESR (SFig. 3). We choose an $\mathrm{EDSR}^{\mathrm{off}}$ tone with half the amplitude of those used for the flip-flop coherence measurements in the main text, and offset in frequency by 5 MHz in order to avoid inducing any resonant spin transitions. For comparison, we also perform reference measurements without the additional $\mathrm{EDSR}^{\mathrm{off}}$ tone. We fit the data (SFig. 3 D-F) with a damped sinusoid $P\exp(-t/\tau^{\mathrm{R}})\sin(2\pi f^{\mathrm{R}}t+\phi)+P_\infty$ (Rabi) and an exponential decay $P\exp(-(\tau/T_2)^\beta)+P_\infty$ (Ramsey and Hahn echo). Here, P is the amplitude, P_∞ is the offset, f^{R} is the frequency, ϕ is the initial phase, t is the duration of the Rabi oscillations and τ^{R} is the Rabi decay time. In Ramsey and Hahn echo decay equation, τ is the total precession time, T_2 is the decay (coherence) time and β is the exponent of the decay.

In SFig. 3 D, we see that the electron Rabi decay time $\tau_{\mathrm{e}}^{\mathrm{R}}$ decreases from 460(73) μs to 69(6) μs when the electric drive is applied simultaneously. The Ramsey coherence time in SFig. 3 E is not affected, but

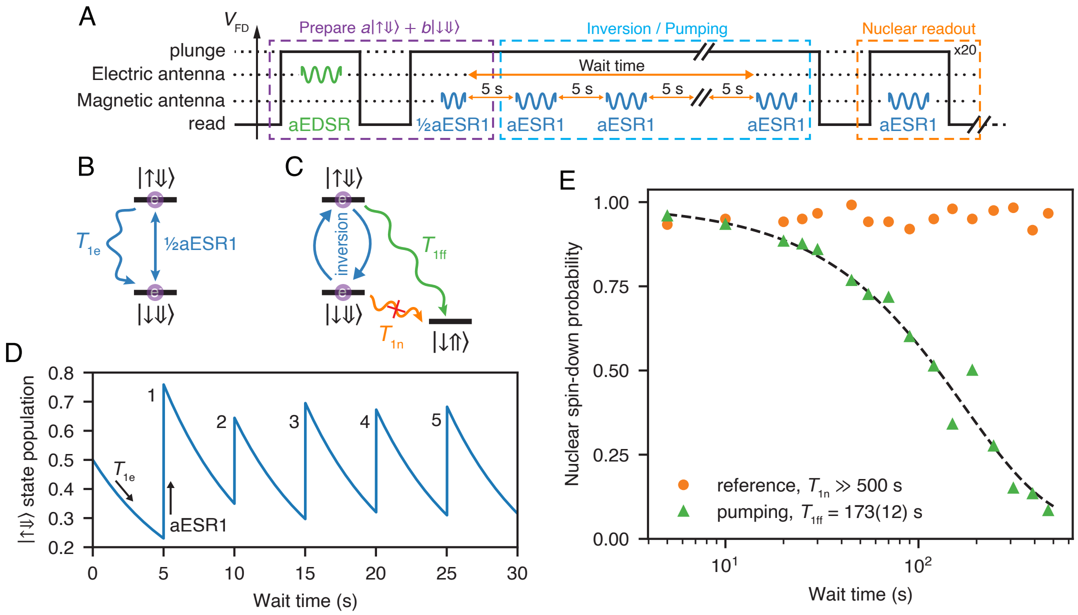

Supplementary Figure 2: **Flip-flop relaxation.** (**A**) The pulse sequence used to measure the flip-flop relaxation time $T_{1\mathrm{ff}}$ first prepares a superposition state of $|{\downarrow\Downarrow}\rangle$ and $|{\uparrow\Downarrow}\rangle$ (purple part). Repeatedly applying aESR1 pulses in a 5 s interval ensures a non-zero population of $|{\uparrow\Downarrow}\rangle$ (blue part). The nuclear decay is measured by measuring the nuclear spin-down probability (orange part). (**B**) We prepare the donor in a superposition $a\,|{\downarrow\Downarrow}\rangle + b\,|{\uparrow\Downarrow}\rangle$ state with $|a|^2 \approx |b|^2 \approx 0.5$. (**C**) By repeatedly inverting the population between $|{\uparrow\Downarrow}\rangle$ and $|{\downarrow\Downarrow}\rangle$ using aESR1 pulses, we keep the excited flip-flop state populated and are able to measure the relaxation process $T_{1\mathrm{ff}}$ via nuclear decay into the $|{\downarrow\Uparrow}\rangle$ state. There is no direct nuclear spin relaxation $T_{1\mathrm{n}}$ since the nucleus is decoupled from the environment. (**D**) The calculated population of the $|{\uparrow\Downarrow}\rangle$ state for the first 30 s of the wait time in the pulse sequence in (**A**). Because of a 5 s-delay between the aESR1 inversion pulses, the population decays due to $T_{1\mathrm{e}}$ relaxation process. We consider $T_{1\mathrm{e}} \approx 6.45$ s, the inversion fidelity of the aESR1 pulses is 98%, and neglect the population decay due to the flip-flop relaxation process in the first 30 s. (**E**) The nuclear spin-down probability dependence on the duration of the wait time in the sequence in (**A**) (green triangles). From the exponential fit (see text), the flip-flop relaxation time is estimated to be $T_{1\mathrm{ff}} = 173(12)$ s. To demonstrate the absence of the nuclear spin relaxation $T_{1\mathrm{n}}$ process, we perform a reference measurement without the aESR1 inversion pulses (orange dots).

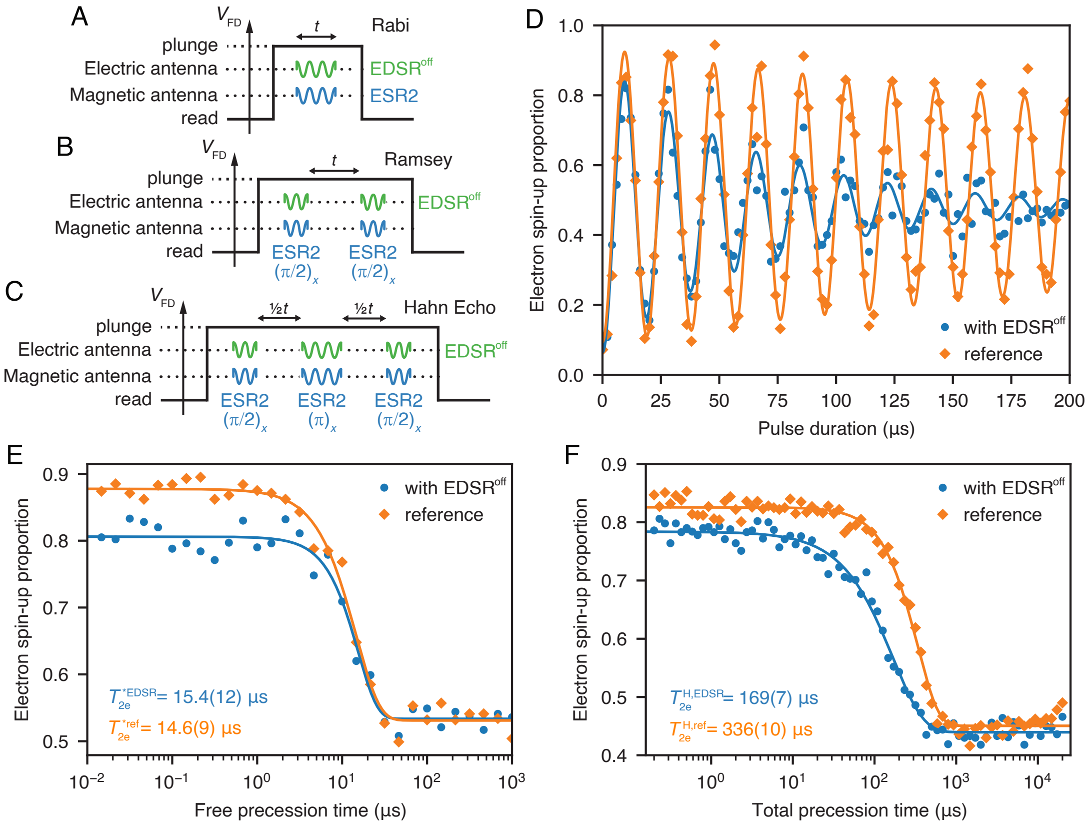

Supplementary Figure 3: **Electron spin coherence.** (**A-C**) Pulse sequences used to measure the electron Rabi (**A**), Ramsey (**B**), and Hahn echo (**C**) decay times while simultaneously applying EDSR$^{\rm off}$ pulses only during the ESR pulses. (**D**) Electron spin Rabi oscillations obtained with (blue dots) and without (orange diamonds) the EDSR$^{\rm off}$ tone. The data are fitted with an exponentially decaying sinusoid $P\exp(-t/\tau_{\rm e}^{\rm R})\sin(2\pi f_{\rm e}^{\rm R}t+\phi)+P_\infty$ revealing that $\tau_{\rm e}^{\rm R}$ decreases from 460(73) μs to 69(6) μs in the presence of the EDSR$^{\rm off}$ tone. (**E-F**) Electron Ramsey (**E**) and Hahn echo (**F**) decays measured with (blue dots) and without (orange diamonds) the EDSR$^{\rm off}$ pulses. The data are fitted with an exponential decay function $P\exp(-(t/T_{2\rm e})^\beta)+P_\infty$ revealing the electron dephasing time $T_{2\rm e}^* = 14.6(9)$ μs without and 15.4(12) μs with the EDSR$^{\rm off}$ pulses. The exponents β^* of the decay are 1.58(17) and 1.91(33), respectively. The Hahn echo time $T_{2\rm e}^{\rm H}$ decreases from 336(10) μs to 169(7) μs when applying the off-resonance EDSR$^{\rm off}$ pulses. The exponent $\beta^{\rm H}$ of the Hahn echo decay also decreases from 1.7(1) to 1.17(7), indicating a potential change in the spectrum of the noise.

we measure a decrease in readout contrast once we apply the $\mathrm{EDSR}^{\mathrm{off}}$ tone. In SFig. 3 F we show the Hahn echo measurement. We find that the $\mathrm{EDSR}^{\mathrm{off}}$ pulse reduces the electron $T_{2\mathrm{e}}^{\mathrm{H}}$ by a factor of two and matches $T_{2\mathrm{ff}}^{\mathrm{H}}$ of the flip-flop qubit. Note that the exponent of the decay changes from 1.7(1) to 1.17(7), when applying $\mathrm{EDSR}^{\mathrm{off}}$ pulses, which indicates a change in the spectrum of the noise experienced by the electron spin [56].

The previous experiments have demonstrated an effect of the $\mathrm{EDSR}^{\mathrm{off}}$ pulse on the coherence of the electron qubit. In the following sections, we take a closer look on the underlying effect. We find that the $\mathrm{EDSR}^{\mathrm{off}}$ drive tone causes a shift in resonance frequencies depending on its on duration and amplitude. Hence, for the measurements where we apply the magnetic and the electric tone simultaneously, the ESR drive becomes off-resonant which leads to the observed decay in the electron Rabi (SFig. 3 D). The π- and $\pi/2$- pulses used in the Ramsey and echo experiment deteriorate and decrease the spin-up proportion and refocusing properties for those measurements.

ESR frequency shifts

To investigate the effect of an $\mathrm{EDSR}^{\mathrm{off}}$ pulse on the ESR resonance frequency, we perform two interleaved ESR spectrum scans around the ESR1 and ESR2 resonances. The first scan is a regular ESR spectrum scan for reference; the second is taken while adding a 5 MHz off-resonant $\mathrm{EDSR}^{\mathrm{off}}$ pulse at the same time as the ESR inversion pulse. The ESR inversion itself is performed using a 9π pulse instead of a simple π pulse, to allow for a longer $\mathrm{EDSR}^{\mathrm{off}}$ pulse and amplify its effects on the ESR resonances. The duration of these inversion pulses is 75 μs for ESR1 and 85 μs for ESR2 (different transmission for the two frequency ranges). For these measurements the magnetic field is set to $B_0 \approx 0.9$ T.

Since most of the spectra are affected by ^{29}Si flips (see S4), we perform multiple repetitions and fit individual resonances with a Gaussian function before averaging (SFig. 4 A-D). Compared to the reference peaks, we find that the resonance is at a higher frequency for both ESR1 and ESR2 frequencies when an $\mathrm{EDSR}^{\mathrm{off}}$ tone is applied at the same time. The ESR resonance is given by the Zeeman splitting and the hyperfine interaction A as $f_{\mathrm{ESR1/2}} \approx \gamma_{\mathrm{e}} B_0 \mp A/2$. As both transitions are shifted towards higher frequencies, we conclude that the frequency shift is caused predominantly by a change in the electron g_{e}-factor [15]. The ESR frequency shifts appear to depend linearly on both the amplitude and the duration of the the off-resonance $\mathrm{EDSR}^{\mathrm{off}}$ tone (SFig. 4 E-F)

NMR frequency shifts

Next we investigate the effects of the EDSR tone on the NMR spectra by measuring the NMR1 and NMR2 spectra and applying the off-resonant $\mathrm{EDSR}^{\mathrm{off}}$ tone right before the NMR pulse. The extracted resonance frequency shifts, amplitudes and line widths as a function of the $\mathrm{EDSR}^{\mathrm{off}}$ pulse duration are shown in SFig. 5 . In SFig. 5 A, we see that the resonances for NMR1 and NMR2 both shift to smaller values. This indicates a reduction in the hyperfine interaction as the NMR resonance frequency is given by $f_{\mathrm{NMR1/2}} \approx A/2 \pm \gamma_{\mathrm{n}} B_0$ (in the electric field range available to our system, the nuclear γ_{n} can be assumed constant). Compared to the ESR spectra, the NMR frequency shifts are at least five times smaller in absolute terms, confirming that the hyperfine shift is weaker than the g_e-factor shift affecting the ESR frequency. For low EDSR drive amplitudes, the NMR frequency shifts are not resolvable in the spectrum scans. As shown in SFig. 5 B-C, the $\mathrm{EDSR}^{\mathrm{off}}$ tone also changes the amplitude and the width of the NMR resonance.

EDSR frequency shifts

Changes of the electron g_{e}-factor caused by a strong electric drive tone also affect the resonance frequency of the flip-flop qubit. To quantify the EDSR frequency shifts, we measure the EDSR resonance frequency after applying an off-resonant $\mathrm{EDSR}^{\mathrm{off}}$ pre-pulse and compare the spectrum to a reference measurement omitting the additional $\mathrm{EDSR}^{\mathrm{off}}$ tone. SFig. 5 D shows the EDSR frequency as a function of the $\mathrm{EDSR}^{\mathrm{off}}$ pre-pulse duration for two different EDSR amplitudes. Contrary to the effect on the ESR and NMR resonances, we find that the frequency shift saturates at longer duration. The saturation time τ_{sat} appears

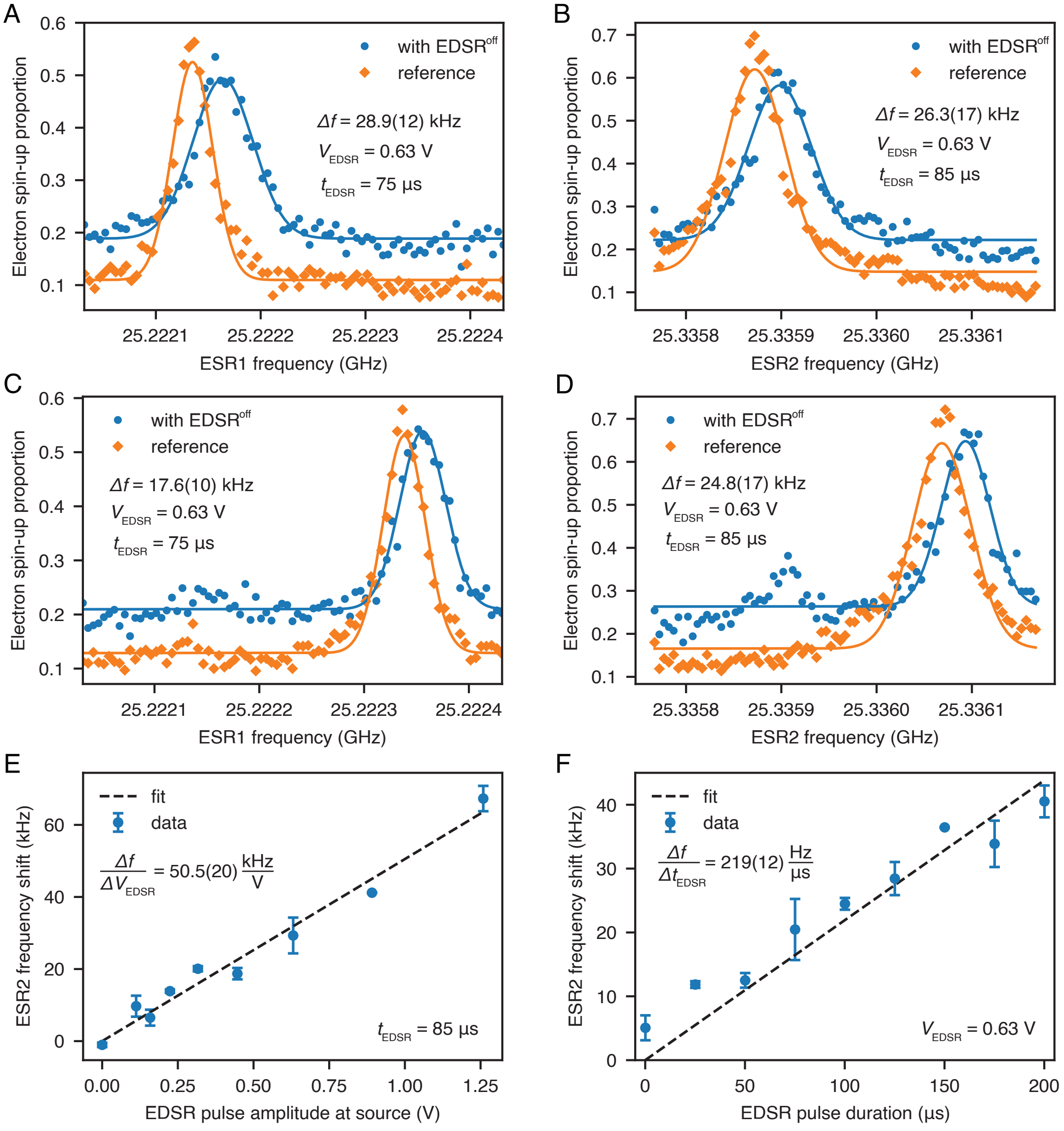

Supplementary Figure 4: **ESR frequency shift.** (**A-D**) ESR spectrum scans averaged over multiple repetitions around the ESR1 (**A, C**) and ESR2 (**B, D**) resonances, measured with (blue dots) and without (orange diamonds) applying an EDSR$^{\rm off}$ pulse simultaneously with the ESR inversion pulse (see text). We show two resonance peaks (**A, C**) for ESR1 and (**B, D**) for ESR2 transitions corresponding to two detected configurations of nearby ^{29}Si nuclear spins. The detected shifts in the resonance frequencies Δf from applying the EDSR$^{\rm off}$ pulse are shown in the corresponding figures. (**E-F**) By changing the amplitude (**E**) and duration (**F**) of the EDSR$^{\rm off}$ pulse, we find a linear increase of the ESR2 frequency shift with $\Delta f/\Delta V_{\rm EDSR} = 50.5(20)$ kHzV^{-1} (**E**) and $\Delta f/\Delta t_{\rm EDSR} = 219(12)$ Hzμs^{-1} (**F**). For the measurement in (**F**), the EDSR$^{\rm off}$ pulse has 0.63 V amplitude at the MW source and its end is aligned to the end of the ESR pulse.

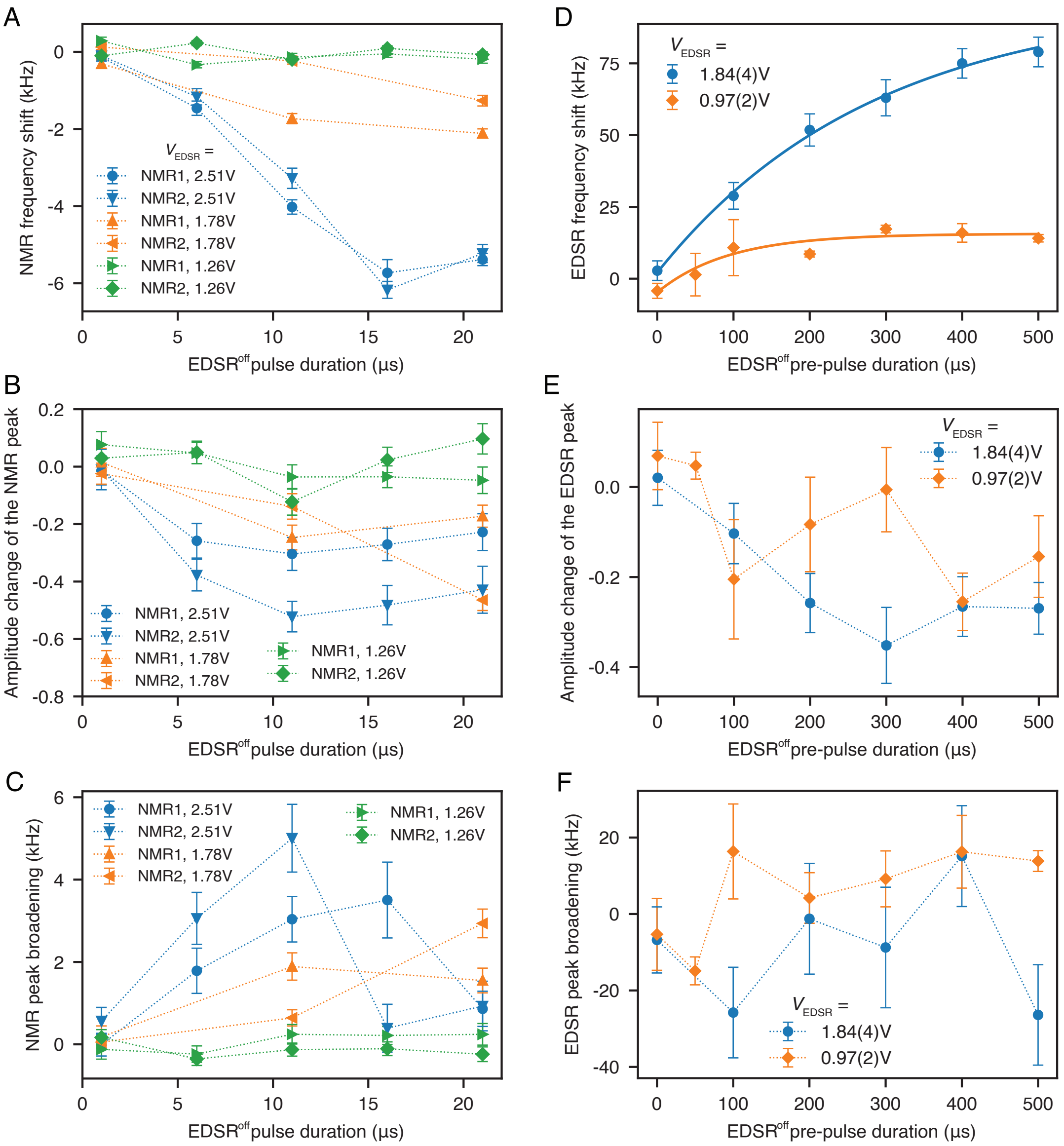

Supplementary Figure 5: **NMR and EDSR frequency shifts.** (**A-C**) Frequency shift (**A**), amplitude change (**B**) and FWHM broadening (**C**) of the NMR1 and NMR2 resonance peaks in the spectrum scans for different durations of the EDSR$^{\rm off}$ pulse. We measure these dependencies for three amplitudes $V_{\rm EDSR}$ = 2.51 V (blue), 1.78 V (orange) and 1.26 V (green) of the EDSR$^{\rm off}$ pulse at the MW source. **(D-F)** Frequency shift (**D**), amplitude change (**E**) and FWHM broadening (**F**) of the EDSR resonance peak in the spectrum scans for different durations of the EDSR$^{\rm off}$ pre-pulse (see text). We measure these dependencies for two amplitudes $V_{\rm EDSR}$ = 1.84(4) V (blue) and 0.97(2) V (orange) of the EDSR pulses at the MW source. The EDSR frequency shifts are fitted with an exponential function $\delta f_{\rm EDSR} = \delta f_{\rm A}(1 - \exp(-t/\tau_{\rm sat})) + \delta f_0$, where the fit parameters are given in STab. 1.

to change for different amplitudes of the EDSR^{off} tone. We fit the dependence with an exponential function of the form $\delta f_{\text{EDSR}} = \delta f_{\text{A}}(1 - \exp(-t/\tau_{\text{sat}})) + \delta f_0$. The fit parameters are given in STab. 1.

Similar to the NMR frequency shifts, the amplitude of EDSR resonance peak also decreases with the pre-pulse duration (SFig. 5 E). However, we do not find any clear duration dependence for the FWHM of the EDSR peak like we see for the NMR peak (SFig. 5 C and F).

EDSR amplitude	δf_{A} (kHz)	τ_{sat} (μs)	δf_0 (kHz)
1.84(4) V	94.8(50)	284(34)	2.1(18)
0.97(2) V	20.4(32)	93(37)	-4.7(28)

Supplementary Table 1: The table shows the parameters extracted from the fits to the EDSR frequency shift data. The fit model is given by $\delta f_{\text{EDSR}} = \delta f_{\text{A}}(1 - \exp(-t/\tau_{\text{sat}})) + \delta f_0$.

S3: Residual ^{29}Si nuclear bath

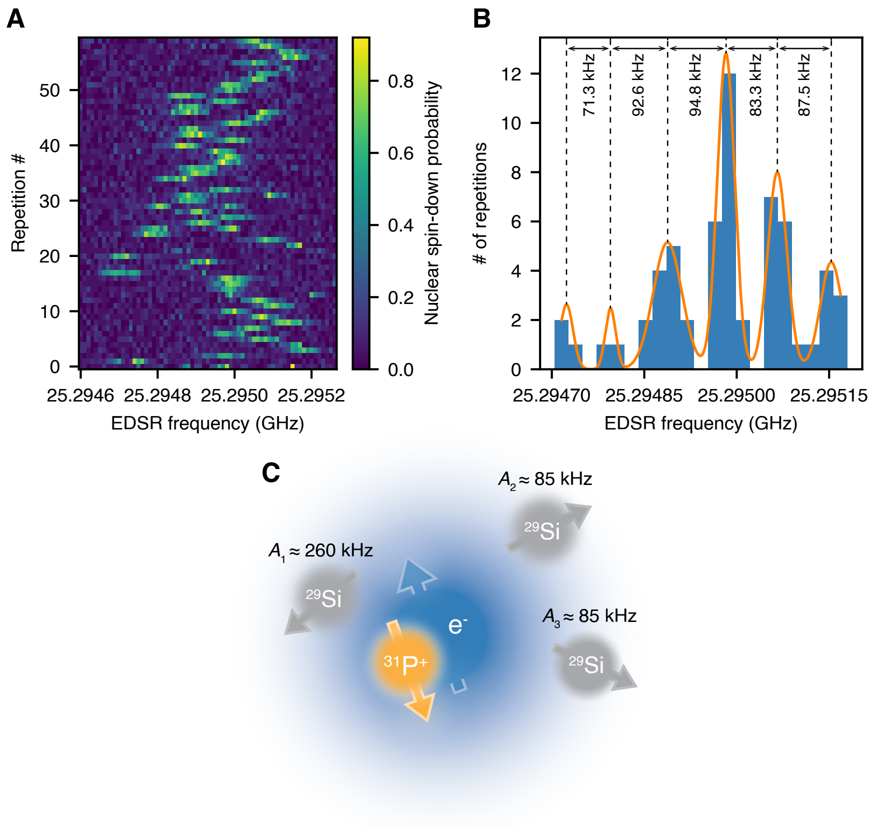

Supplementary Figure 6: **^{29}Si nuclear spin flips.** **(A)** Tracking the EDSR resonance while applying a NMR pulse at the ^{29}Si resonance frequency reveals discrete jumps in the flip-flop frequency. **(B)** The histogram shows the extracted EDSR resonance frequencies from **(A)**. We find six clusters of frequencies that are $\approx 19 - 55$ kHz wide (FWHM) and separated by $\approx 70 - 95$ kHz. **(C)** The spectra can be reproduced with at least three hyperfine coupled ^{29}Si nuclei (see text for details).

The isotopically enriched ^{28}Si epitaxial layer has a residual ^{29}Si concentration of 730 ppm. With such value, one may expect of order 10 ^{29}Si atoms within the Bohr radius of a ^{31}P donor [57], although

only a small subset of them may possess a strong enough hyperfine coupling to the donor-bound electron to result in a visible effect on the resonance spectrum. We found that the donor under study in this paper is significantly coupled to (at least) three proximal ^{29}Si atoms. The ^{29}Si isotope has spin $1/2$ and a gyromagnetic ratio $\gamma_{\text{Si29}}/2\pi = 8.465$ MHz/T and can couple to the donor-bound electron via the hyperfine interaction A_{Si29}. In a semiclassical picture, this interaction can be considered as an additional magnetic field, which shifts any resonance frequency f_{res} depending on the coupling strength and the orientation of spins of the ^{29}Si atoms:

$$f_{\text{res}} = f_{\text{res},0} \pm \sum_i \frac{A_{\text{Si29,i}}}{2}. \tag{S.2}$$

Long-term EDSR spectrum measurements reveal that the EDSR resonance frequency randomly switches between 6 different values separated by $\approx 70 - 95$ kHz on a timescale that fluctuates between seconds and hours. To speed up the measurement and confirm that the frequency jumps are caused by the surrounding bath of ^{29}Si, we apply a NMR π-pulse at the resonance frequency of the ^{29}Si nuclei with the donor ionized, $\gamma_{\text{Si29}} B_0/2\pi \approx 7.644$ MHz ($B_0 = 0.9$ T for this experiment). In the absence of a hyperfine-coupled electron, this frequency is the same for all ^{29}Si atoms and the NMR π-pulse will flip the ^{29}Si spin configuration. In SFig. 6 A, we plot 60 EDSR spectra taken while applying a ^{29}Si NMR pulse in between repetitions. We see that the EDSR resonance changes between most of the repetitions. A reference scan omitting the NMR pulses shows less frequency switching which indicates that the frequency jumps are caused by ^{29}Si spin flips in the proximity of the qubit.

We extract the instantaneous EDSR resonance frequency by fitting individual spectra with a Gaussian function and show the histogram of the values in SFig. 6 B. We find a cluster of six EDSR frequencies separated by $\approx 70 - 95$ kHz and with a full-width-half-maximum of $\approx 19 - 55$ kHz. According to Eq. (S.2), we conclude that the qubit is hyperfine coupled to at least three nearby ^{29}Si atoms which should in principle result in $2^3 = 8$ different frequencies (see SFig. 6 C). We can reproduce the coupled system if we assume that two of the hyperfine couplings are within 10 kHz of each other, for instance $A_1 = 260(20)$ kHz and $A_2 \approx A_3 = 85(10)$ kHz.

The persistent frequency jumps require tracking of the instantaneous resonance frequency. Hence, we are forced to regularly perform frequency scans and update all relevant frequencies during and between measurements.

S4: Gate Set Tomography and Randomized Benchmarking

The gate set tomography (GST) protocol provides a detailed, calibration-free and self-consistent characterization of quantum gates [58, 59, 28]. It quantitatively identifies gate errors and allows to correct some of them, e.g. under- or over-rotation of the qubit. The gate set under investigation consists of a $X_{\pi/2}$, $Y_{\pi/2}$ and an identity I gate. The $X_{\pi/2}$ and $Y_{\pi/2}$ gates are implemented as 90°-phase shifted, resonant EDSR $\pi/2$-pulses. The duration of 3.04 μs is limited by the maximum amplitude of the EDSR pulse of 2.59(6) V that does not destabilize the device (see S9). The I gate is implemented as a 1 μs long delay without applying any EDSR tone. The delay is chosen to be shorter than $T^*_{2\text{ff}} = 4.09(88)$ μs of the flip-flop qubit to limit the overall dephasing error during GST.

We measure the outcome of 448 GST circuits, each consisting of an initialization into $|{\downarrow}{\Uparrow}\rangle$ state, a combination of gates $\in \{X_{\pi/2}, Y_{\pi/2}, I\}$ of varying length (up to maximum eight gates in the repeated germ sequences [28, 60]) and a measurement of the nuclear spin $|{\Downarrow}\rangle$ proportion. The initial circuit sequences characterize state preparation and measurement error of the gate set, whereas later circuits use error amplification techniques to map out the fidelity of the gates. The ideal measurement outcome of each circuit is a nuclear spin $|{\Downarrow}\rangle$ proportion of either 0, 0.5 or 1; a deviation from these values indicates gate errors.

Each GST circuit is repeated 100 times to collect output statistics, and the entire sequence of 448 circuits is repeated twice, thus yielding 200 measurement shots for each circuit. The measurement results

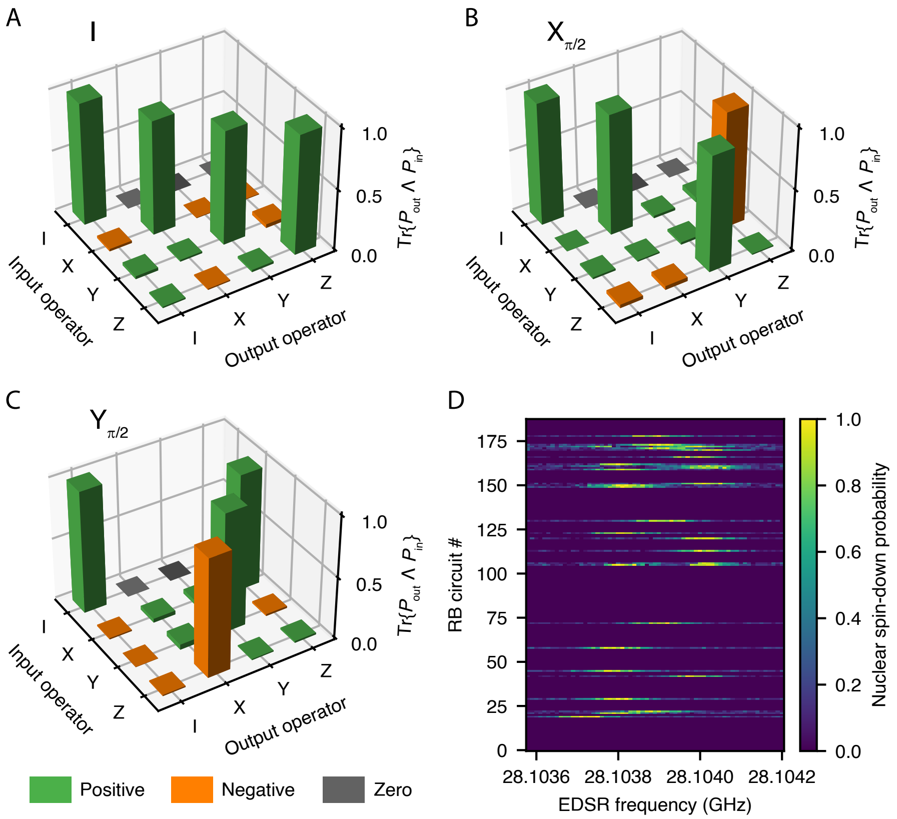

Supplementary Figure 7: **GST process matrices and RB spectrum checks. (A), (B) and (C)** GST-estimated process matrices for the flip-flop qubit logic gates: **(A)** identity I, **(B)** $\pi/2$ rotation around X, $X_{\pi/2}$, and **(C)** around Y, $Y_{\pi/2}$. **(D)** We track and update the EDSR resonance frequency before and after each measurement of the GST and RB circuit to reduce the impact of ^{29}Si spin flips. Here we show such a frequency check during the RB experiment. Blue lines indicate a stable configuration of the surrounding spins.

are then fitted self-consistently by PyGSTi [61]. The GST analysis determines single-qubit gate fidelities between 97.5% – 98.5% (see Table 2).

In addition, GST provides information about the contribution of different types of errors affecting the qubit control, which include coherent (under-/over-rotation of the qubit), stochastic (decoherence-like), affine (relaxation-like), and all other errors. While the majority of the infidelity is dominated by decoherence (between 36% and 77% of the total error), we still diagnose a small under-rotation of $0.8^\circ - 1.31^\circ$ for $X_{\pi/2}$ and $Y_{\pi/2}$ gates (see Table 2). The affine errors are minor for our flip-flop qubit, as expected from the long flip-flop relaxation time shown in S2. For a further description of the error types that are analyzed by GST, see Refs. [62, 41]. GST also estimates the state preparation and measurement (SPAM) probability, i.e. the fidelity of the flip-flop qubit initialization into the $|\downarrow\Uparrow\rangle$ state, yielding $F_{\text{SPAM}} = 91.97\%$.

The reconstructed process matrices for the gate set are shown in SFig. 7 . The GST report also reveals model violations due to the presence of non-Markovian dynamics. Non-Markovianity is present in our system as a result of random ^{29}Si spin flips and the shift in resonance frequency from applying high-power EDSR pulses (see S3 and S4). To minimize the first effect, we check the EDSR resonance frequency before and after every GST sequence, and remeasure the sequence if the resonance frequencies don't match. However, we are still sensitive to ^{29}Si spin flips within the measurement sequence itself and during the time it takes to upload the pulse sequence to the AWG.

Gate	Pulse duration	Rotation angle	Coherent errors	Stochastic errors	Affine errors	Fidelity (average)
I	1 μs delay	0.0085π	12%	77%	9.2%	97.5%
$X_{\pi/2}$	3.04 μs	0.4955π	33%	50%	11%	98.2%
$Y_{\pi/2}$	3.04 μs	0.4927π	49%	36%	2.4%	98.5%

Supplementary Table 2: This table shows partial results from the GST analysis for the flip-flop gate set. The errors are presented as a percentage of the total error.

We additionally perform randomized benchmarking (RB), a comparatively simple, SPAM-insensitive characterization method that provides the average single qubit gate fidelity [63]. For a typical RB pulse sequence the qubit is first initialized in the $|\downarrow\Uparrow\rangle$ state. Then we apply a random sequence of Clifford gates of length m up to a maximum of 65. Before reading out the nuclear spin state, we apply a final Clifford gate that returns the qubit back to $|\downarrow\Uparrow\rangle$. Any deviation from $|\downarrow\Uparrow\rangle$ implies errors in the Clifford gates. By varying the sequence length and measuring the decay constant, we can deduce the average gate errors of the Clifford gates. The Clifford gates are constructed from a combination of native $X_\pi, Y_\pi, X_{\pi/2}, Y_{\pi/2}$ gates. We used a set of gates from the open-source software PyGSTi where the average number of native gates per Clifford is ≈ 2.233.

In light of the results obtained earlier from GST, we adjusted the EDSR pulse duration of the $X_{\pi/2}, Y_{\pi/2}$ gates to account for the under-rotation detected in that experiment (compare STab. 2 and STab. 3). As for the GST experiment, we sandwich every RB sequence between EDSR spectra and remeasure RB sequences if the resonance changes due to ^{29}Si spin flips. We find an average Clifford gate fidelity $\mathcal{F}_{\text{C}} = 96.4(5)\%$, which corresponds to an average native gate fidelity $\mathcal{F}_{\text{1Q}} = 98.4(2)\%$. The results obtained from RB are in good agreement with the GST fidelities.

In an attempt to account for PIRS effects, we adjusted the drive frequency with increasing RB pulse length to follow the measured exponential dependence (see S3). Unfortunately, we did not find an increase in the average gate fidelity of those experiments in comparison to just using single-frequency sine pulses described above.

S5: Capacitive triangulation of the donor location

The Phosphorus donors are implanted within a 100 nm×90 nm region underneath the FD gate and in close proximity to the SET and magnetic antenna. This means that the exact location of the specific

Gate	Pulse duration
X	6.13 μs
Y	6.13 μs
$\pm X_{\pi/2}$	3.073 μs
$\pm Y_{\pi/2}$	3.087 μs

Supplementary Table 3: Native gates and their respective EDSR pulse durations used in the RB experiment.

donor which we used as qubit is a priori unknown.

To narrow down its possible location, we use a triangulation method based on comparing the capacitive couplings between the donor-bound electron and several electrostatic gates in the device [64, 12].

We start by measuring charge stability diagrams around the donor transition using different gate electrodes (see SFig. 8 A-B as an example for the LS, RS and FD gates). The white dotted line is the so-called donor charge transition, where the electrochemical potentials of the donor and the SET are aligned. This means that the electrostatic potential $V(\vec{r}_0, V_{\mathrm{FD}}, V_{\mathrm{RS}}, V_{\mathrm{LS}}, \dots)$ at the donor location $\vec{r}_0$ is kept constant along the transition and the gate voltages in SFig. 8 A must satisfy the relation

$$\frac{\partial V(\vec{r}_0, V_{\mathrm{FD}}, V_{\mathrm{LS}}, V_{\mathrm{RS}}, \dots)}{\partial V_{\mathrm{FD}}}\delta V_{\mathrm{FD}} + \frac{\partial V(\vec{r}_0, V_{\mathrm{FD}}, V_{\mathrm{LS}}, V_{\mathrm{RS}}, \dots)}{\partial V_{\mathrm{LS}}}\delta V_{\mathrm{LS}} = 0, \tag{S.3}$$

where δV_{FD} and δV_{LS} are the respective gate voltage changes along the transition. This relation can be rewritten as

$$\frac{\delta V_{\mathrm{FD}}}{\delta V_{\mathrm{LS}}} = -\frac{\partial V(\vec{r}_0, V_{\mathrm{FD}}, V_{\mathrm{LS}}, \dots)}{\partial V_{\mathrm{LS}}} \Bigg/ \frac{\partial V(\vec{r}_0, V_{\mathrm{FD}}, V_{\mathrm{LS}}, \dots)}{\partial V_{\mathrm{FD}}}, \tag{S.4}$$

where the ratio between the gate capacitances (left hand side) represents the slope of a transition, i.e. s_{LS}, in the charge stability diagram in SFig. 8 A. In the same way, we determine the slopes for the remaining gate combinations (see SFig. 8 B for RS and FD gates, the rest of the combinations are available in a public data repository).

Next, we perform simulations of the electrostatic potential landscape of the device using the COMSOL software package, to calculate the right hand side of Eq. S.4 for positions $\vec{r}$ within the 200 nm$\times$200 nm area around the implantation window (see SFig. 8 C). In these simulations, we model the device according to our design layout and consider the 2DEG underneath the SET as a 1 nm thick metallic layer at the Si/SiO_2 interface, with lateral dimension reflecting those of the SET.

Having obtained the right hand side slopes $s_g^{\mathrm{sim}}(\vec{r})$ in Eq. S.4 for all measured gates $g \in \{\mathrm{LS}, \mathrm{RS}, \dots\}$, we compare them to the experimental values s_g using a least-squares estimate [65, 12] as

$$P(\vec{r}) = N \exp\left[-\frac{1}{2}\sum_{g\in\{\mathrm{LS},\mathrm{RS},\dots\}}\left(\frac{s_g^{\mathrm{sim}}(\vec{r}) - s_g}{\sigma_g}\right)^2\right], \tag{S.5}$$

which returns the maximum probability density $P(\vec{r_0})$ at each position $\vec{r_0}$, where the difference between simulated and measured slopes is minimal. N is a normalization factor and σ_g is the standard deviation error of the measured slope s_g for gate g which we define as

$$\sigma_g = 1 + \frac{1}{s_g^2}. \tag{S.6}$$

In this way we give more weight to the gates that have larger slopes, i.e. stronger capacitive coupling to the donor, since their effect can be estimated more reliably. The gates with smaller slopes are less reliable as they are strongly screened by the nearby gates and the 2DEG, and hence provide less accurate information about the donor position.

As seen in SFig. 8 C, the capacitive model yields two regions where the ^{31}P donor could be located: at the bottom-left corner of the device, i.e. under the TG and near the LB gates, and underneath the tip of

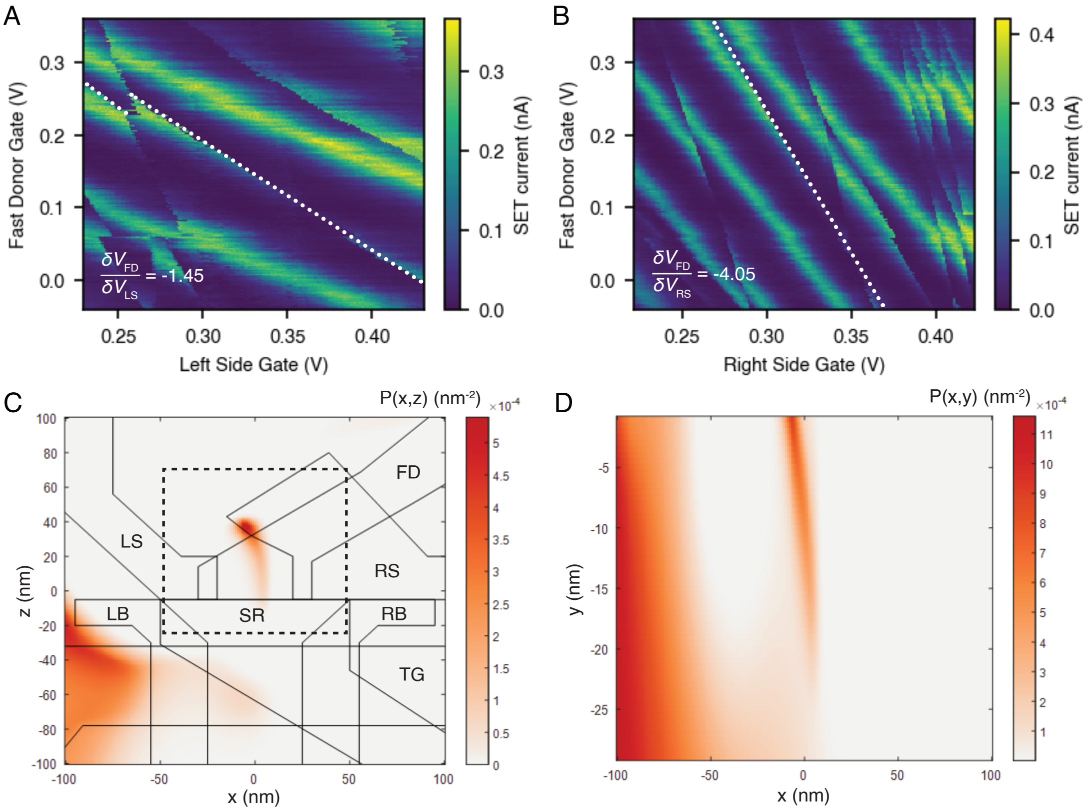

Supplementary Figure 8: **Donor triangulation.** (**A-B**) Charge stability diagrams around the donor transition (white dotted line) using FD, LS and RS gates with the respective slopes of the transition shown in the bottom left corner. (**C-D**) Probability density distribution of the donor location in-plane of the substrate (**C**) and perpendicular to it (**D**). The distribution is calculated by comparing measured and simulated slopes of the donor transition (see Eq. S.5 for details). The dashed black region in (**C**) shows the implantation window in the device. We find a high probability at the tip of the RS gate.

the RS gate. It is very unlikely that the donor is in the first region, since the capacitive coupling of the TG and LB gates to the donor-bound electron is small and this region is outside of the implantation window (black dashed region in SFig. 8 C). We thus ignore this region and consider the donor to be under the tip of the RS gate, slightly behind the FD gate (electric antenna). This location may explain the linear increase of the hyperfine interaction with the FD gate voltage (Fig. 3 B of the main text) instead of the expected decrease, since we increase the electron wavefunction overlap with the donor nucleus when applying a positive voltage on the FD gate [15]. Further possible explanations for an increase in hyperfine coupling with positive FD gate voltage might involve a shallow (< 3.2 nm) depth of the donor under the Si/SiO_2 interface. This would cause a strong electron wavefunction distortion by the interface barrier [66]. Strain at the donor location from the difference in the thermal expansion coefficients of the aluminum gates and the silicon substrate is another mechanism that can lead to a distortion of the electron wavefunction and a positive hyperfine tunability [67, 15, 16, 12]. To exactly identify the contribution from each mechanism would require additional investigation, for example, by using atomistic tight-binding simulations of the hyperfine coupling that include electric fields and strain in the vicinity of the expected donor location from the additional COMSOL simulations [13, 15, 64]. The capacitive triangulation method also provides information about the vertical (donor depth, y-axis) location of the donor (see SFig. 8 D). However, due to the planar gate layout of the donor device, the sensitivity (and hence the precision) of this method in the y-direction is low, which is why we find a large range of high probability density spanning almost 20 nm depth in SFig. 8 D.

S6: EDSR drive amplitude calibration

Assuming the electric field at the donor is proportional to the voltage applied to the FD gate, we can rewrite the equation from the main text as

$$f^{\text{ff}}_{\text{Rabi}} = \frac{1}{2}\frac{\partial A(E)}{\partial E}E_{\text{ac}} = \frac{1}{2}\frac{\partial A(V_{\text{FD}})}{\partial V_{\text{FD}}}\Delta V_{\text{FD}}, \tag{S.7}$$

where $\frac{\partial A(V_{\text{FD}})}{\partial V_{\text{FD}}}$ is the hyperfine tunability with the applied voltage to the FD gate and ΔV_{FD} is the amplitude of voltage oscillations on the FD gate during the EDSR pulse, which we will call the EDSR drive amplitude. The amplitude of these oscillations can be further presented as $\Delta V_{\text{FD}} = \alpha V_{\text{MW}}$, where V_{MW} is the peak-to-peak amplitude of EDSR pulse at the MW source and α is a coefficient representing the attenuation of the EDSR drive amplitude between the MW source and the tip of the electric antenna. The attenuation of the EDSR drive amplitude occurs in the coaxial cables, including a 10 dB attenuator at the 4 K stage and a diplexer at the mixing chamber, at the PCB and the electric antenna itself. As a result, Eq. S.7 becomes

$$f^{\text{ff}}_{\text{Rabi}} = \frac{1}{2}\frac{\partial A(V_{\text{FD}})}{\partial V_{\text{FD}}}\alpha V_{\text{MW}}. \tag{S.8}$$

Comparing the slopes of the measured Rabi frequencies f^{ff}_{R} as a function of microwave power (Fig. 3 A) to the DC Stark shift of the hyperfine coupling (Fig. 3 B) yields the following conversion factor between DC and AC signals (or line attenuation)

$$\alpha_{\text{EDSR}} = 2\frac{\partial f^{\text{ff}}_{\text{R}}}{\partial V_{\text{FD}}}\Bigg/\frac{\partial A(V_{\text{FD}})}{\partial V_{\text{FD}}} = 0.125(7) \equiv -18.1(5)\text{ dB}, \tag{S.9}$$

where the confidence interval in the brackets represents a standard deviation.

We verify this result by an independent calibration of the MW to low-frequency conversion factor based on comparing the effect of a 100 Hz square wave and a 28 GHz MW sinusoid on the broadening of the SET Coulomb peak (along the red dashed line in SFig. 9 A). Supplementary Figures 9 B,D show the splitting of the Coulomb peak as a function of the peak-to-peak amplitude $V^{100\text{Hz}}_{\text{pp}}$ of the 100 Hz square wave, where we average the SET current signal for 1 s at every gate voltage point. We fit the data to two Gaussian functions and determine the Coulomb peak splitting $\Delta V_{100\text{Hz}}$ as the distance between their

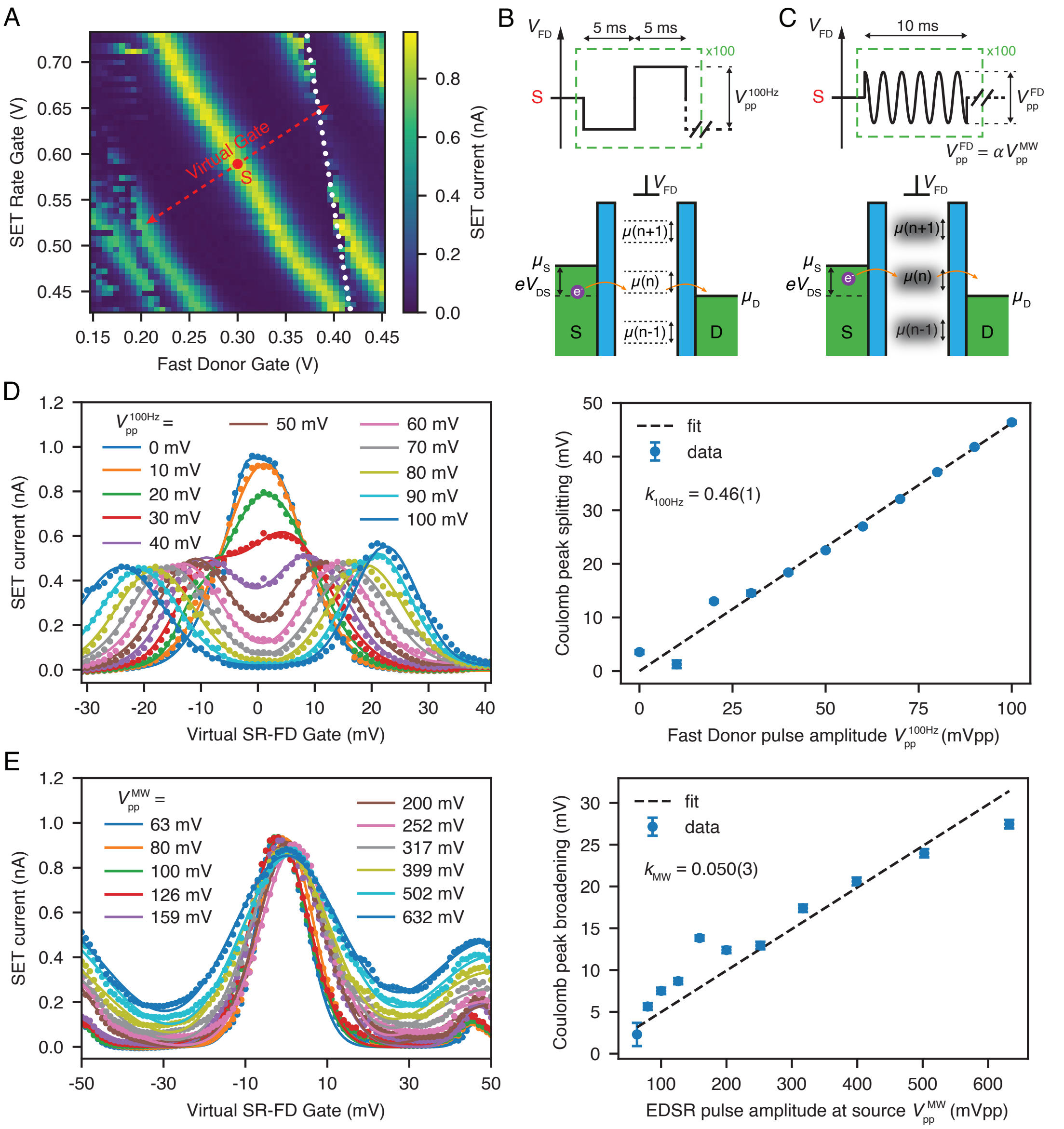

Supplementary Figure 9: **EDSR drive amplitude calibration.** (**A**) The charge stability diagram shows the SET Coulomb peak next to the donor charge transition (white dotted line) used for the calibration procedure outlined in the text. The SET current peak is measured along the red dashed line. (**B**) A 100 Hz square wave pulse periodically splits the electrochemical potential of the SET island in two levels that are occupied half the time. (**C**) A high-frequency EDSR tone applied to the FD gate smears out and broadens the electrochemical potential. (**D**) SET current as a function of the amplitude of the square wave depicted in **B**. The current peak splits in two upon increasing the amplitude, since we average the SET current over many periods of the square wave. We fit two Gaussians to the Coulomb peak doublets to extract the peak splitting as a function of the amplitude of the 100 Hz square wave. (**E**) The MW tone (panel **C**) results in a broadening rather than a splitting of the SET current peak. We fit three Gaussians to the Coulomb peaks to determine the dependence of the central Coulomb peak broadening on the amplitude of the EDSR pulse at the MW source. Comparing the effects of the 100 Hz square wave and the MW tone allows to infer the drive amplitude $V_{\mathrm{pp}}^{\mathrm{MW}}$, since the low-frequency square wave amplitude $V_{\mathrm{pp}}^{\mathrm{100Hz}}$ is accurately known.

mean values. We obtain a linear dependence of $\Delta V_{100\mathrm{Hz}}$ on the amplitude $V_{\mathrm{pp}}^{100\mathrm{Hz}}$ of the FD pulse (see SFig. 9 D), described by

$$\Delta V_{100\mathrm{Hz}} = k_{100\mathrm{Hz}} V_{\mathrm{pp}}^{100\mathrm{Hz}}, \tag{S.10}$$

where $k_{100\mathrm{Hz}} = 0.46(1)$. The splitting $\Delta V_{100\mathrm{Hz}}$ is almost half of $V_{\mathrm{pp}}^{100\mathrm{Hz}}$, since we apply the square wave pulse to FD only, but scan across the Coulomb peak in the SR direction as well (SFig. 9 A) by means of a 'virtual gate'.

The Coulomb peak broadening due to a microwave tone (applied at the flip-flop resonance frequency in order to calibrate at the frequency of interest, although the spin dynamics has no bearing on the experiment) is shown in SFig. 9 C,E. We extract the broadening as the FWHM ΔV of the middle peak by fitting the data to three Gaussian peaks. The excess broadening due to the EDSR tone is then calculated as $\Delta V_{\mathrm{MW}} = \sqrt{\Delta V^2 - \Delta V_{\mathrm{ref}}^2}$, by subtracting a reference value recorded without the drive tone applied [19]. The linear dependence of the Coulomb peak broadening on the EDSR pulse amplitude $V_{\mathrm{pp}}^{\mathrm{MW}}$ at the MW source, shown in SFig. 9 E, is described by

$$\Delta V_{\mathrm{MW}} = k_{\mathrm{MW}} V_{\mathrm{pp}}^{\mathrm{MW}}, \tag{S.11}$$

where $k_{\mathrm{MW}} = 0.050(3)$. Deviations of the data from the linear dependence in SFig. 9 E are mainly attributed to thermal heating of the device during high-amplitude EDSR pulses, which contributes to the broadening of the Coulomb peak and leads to an increase of the offset of the current trace, which is currently poorly understood.

Equating Eqs. S.10 and S.11 and substituting $V_{\mathrm{pp}}^{100\mathrm{Hz}} = \tilde{\alpha} V_{\mathrm{pp}}^{\mathrm{MW}}$ we derive the conversion factor between MW and 100 Hz signals to:

$$\tilde{\alpha} = \frac{k_{\mathrm{MW}}}{k_{100\mathrm{Hz}}} = 0.107(7) \equiv -19.4(5)\ \mathrm{dB}. \tag{S.12}$$

The independently determined conversion factor agrees well with the one extracted from Rabi and hyperfine measurements. The slight discrepancy of 1.3 dB can be explained by a change in lever arm of the FD gate to the SET island and the donor itself, i.e. the ratio between DC and AC electric field can be different at both locations. Thermal broadening and rectification effects due to high power electric signals and changes in the resonance frequency due to spin flips of nearby ^{29}Si nuclei in combination with a frequency-dependent line attenuation can also influence the estimate of the conversion factors.

The good numerical agreement between the two estimates affirms that the Rabi drive strength is given by the Stark shift of the hyperfine coupling and that hyperfine-mediated EDSR is the driving mechanism for the flip-flop qubit.

S7: 3D microwave cavity design for nuclear hyperpolarization

As described in the main text, the coherent electrical drive of the flip-flop transition demonstrated here suggests a very efficient way to produce nuclear hyperpolarization in bulk ensembles. This, however, requires the design of a peculiar resonator, capable of delivering both electric and magnetic fields at microwave frequencies. Here we describe a possible design for such resonator.

The cavity is formed from a 2-mm-thick block of copper suspended in a rectangular copper cavity on insulating plastic screws and washers made from polyether ether ketone (PEEK), see SFig. 10 . The copper block perturbs the cavity modes and concentrates the electric and magnetic fields in the regions between the block and the cavity walls (SFig. 11). We focus on the loaded TE_{101} mode, which has a transverse electric field component centered in the cavity and a magnetic field profile concentrated towards the outer edges. A silicon sample (1.5 mm $\times$ 2 mm $\times$ 0.5 mm) is placed on top of the block and towards the edge (see SFig. 10), in a region with both strong magnetic and electric fields. In addition to concentrating the AC electric and magnetic fields, the metal block provides a means to apply a DC electric field for inducing a linear Stark effect (see discussion in the main text). To DC bias the block, an electrode is attached at its end, in a region corresponding to a node of the AC electric field. Connecting the electrode at this point ensures that any microwave losses through the DC bias port are minimized.

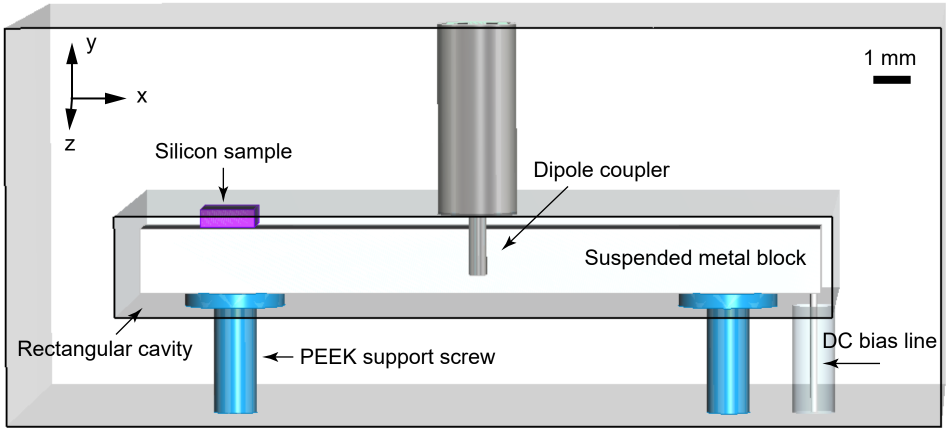

Supplementary Figure 10: **3D model of the loaded rectangular cavity with DC voltage bias line.** Rectangular microwave cavity loaded with a DC-biased suspended metal block made from copper. A cut-plane is taken along the z-axis (i.e. the xy-plane) inside the rectangular cavity to reveal the block, screws and bias electrode. The PEEK screws (metric size M1.6) are insulating and extend 1 mm inside the metal block (not shown). The separation between the top of the block and the rectangular cavity is 0.6 mm.

Applying a voltage to the electrode will generate a uniform DC electric field between the block and the (grounded) rectangular cavity.

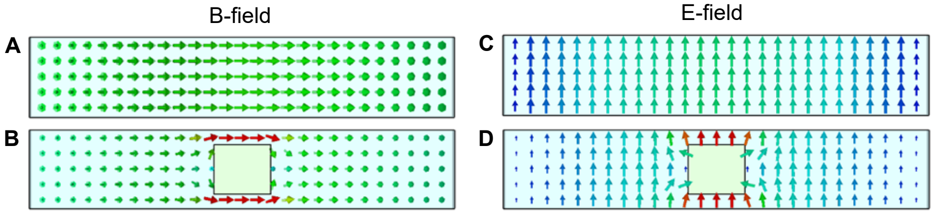

Supplementary Figure 11: **Enhancing the B and E fields of a TE_{101} mode with a suspended metal block.** Magnetic field (**A-B**) and electric field (**C-D**) profiles of the TE_{101} mode without (**A, C**) and with (**B, D**) the suspended copper block. The plots show a cut-plane along the x-axis (i.e. the yz-plane), see SFig. 10 for a depiction of the axes. The colorbar (not shown) is identical in both of the B and E field plots.

We simulate the cavity with the software package CST Studio Suite and use the room-temperature values for the copper conductivity ($\sigma = 5.8 \times 10^7$ S/m) and PEEK dielectric microwave loss tangent ($\tan\delta = 0.003$), so as to provide worst-case values for the expected losses. We find an internal quality factor of $Q_\mathrm{i} = 1150$, with contributions from dielectric losses in the PEEK support screws and washers ($Q_\mathrm{id} = 3600$) and from surface losses in the metal components ($Q_\mathrm{im} = 1700$). When we allow for radiation losses through the DC bias port, the quality factor reduces slightly to $Q_\mathrm{i} = 900$, indicating that the introduction of the DC bias has a negligible impact on the TE_{101} cavity mode loss ($Q_\mathrm{ib} = 4140$). We use a dipole coupler to probe the cavity with microwaves via an external transmission line and choose an external coupling quality factor of $Q_\mathrm{c} = 200$, set by varying the length of the dipole coupling element pin, which results in a loaded quality factor of $Q_\mathrm{l} \approx 165$.

We choose a loaded TE_{101} resonance frequency of $\omega_0/2\pi = 9.95$ GHz (in the microwave X-band), providing a bandwidth for this mode of 60 MHz. This bandwidth is sufficient to cover both the flip-flop transition frequency and one of the hyperfine electron spin resonance (ESR) lines, which have a maximum

separation of 65 MHz at 9.95 GHz ($B_0 \approx 0.35$ T). This allows the simultaneous driving and measurement of both the flip-flop and ESR transition required in the proposed DNP scheme.

In SFig. 12 we show the electric and magnetic AC field strengths for an applied microwave input power of 50 W, a typical power used in commercial ESR systems. We find a magnetic field strength exceeding 1 mT (taking into account the rotating wave approximation) over the sample volume and an electric drive exceeding 200 kV/m inside the silicon. Such an AC electric drive, in combination with an applied DC electric field, could produce Rabi frequencies of order 5 kHz [20] on the flip-flop transition – sufficient to hyperpolarize the ^{31}P nuclear spin using the DNP scheme proposed in the main text in less than a second (The exact value depends on internal fields and strain in the sample).

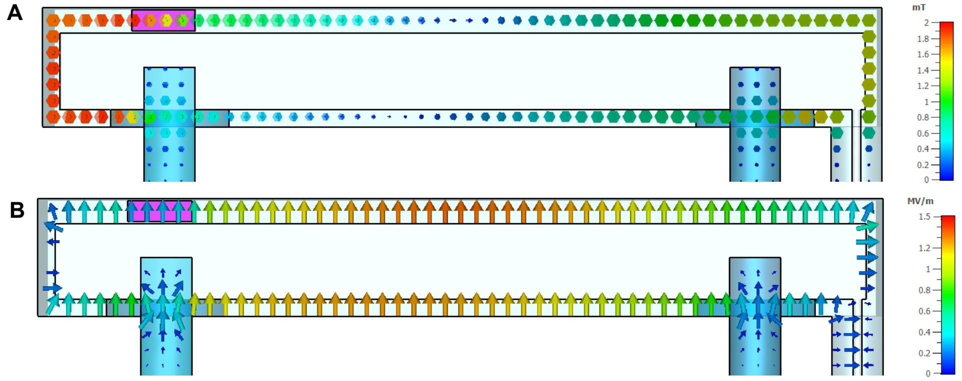

Supplementary Figure 12: **B and E field distribution of the TE$_{101}$ mode.** Magnetic field (**A**) and electric field (**B**) profiles on resonance with the loaded TE$_{101}$ mode at $\omega_0/2\pi = 9.95$ GHz and with an input power of 50 W. Profiles are plot on a cut-plane along the z-axis (i.e. the xy-plane) in the middle of the cavity (see SFig. 10 for depiction of axes).

S8: High donor implantation dose

For a $^{31}P^{+}$ implantation fluence of 1.4×10^{12} atoms/cm^2, we expect ≈ 40 donors in the implantation window at an average depth of $\approx 6.8(33)$ nm below the SiO_2/Si interface. The high donor concentration increases the probability of finding a donor in a convenient location, but reduces the range of gate voltages that can be applied without affecting the charge state of nearby donors.

For a flip-flop qubit, the best gate performance and the most convenient multi-qubit coupling strategy are achieved in the high electric dipole regime, where an electron is significantly displaced from the donor atom [13]. Achieving this regime requires applying large voltage swings to the gates that control the donor potential.

In the present device, we prioritized having a high chance of finding a donor in a convenient location within the device. For this purpose we engineered a $^{31}P^{+}$ implantation fluence of 1.4×10^{12} atoms/cm^2, from which we expect ≈ 40 donors in the implantation window at an average depth of $\approx 6.8(33)$ nm below the SiO_2/Si interface. The resulting charge stability diagram is shown in SFig. 13. It reveals numerous charge transitions – breaks in the straight pattern of SET current peaks – consistent with the large number of implanted donors. The charge transition corresponding to the donor used for the present experiment is shown encased in the red dashed rectangle. The presence of other donors limits the Fast Donor Gate voltage range around the transition to less than ± 200 mV. Crossing other donor transitions would destabilize the electrostatic landscape of the device and perturb the operation of the qubit. Under such limitations, it was not possible to reach the high electric dipole regime.

Future devices will adopt the deterministic single-ion implantation method recently demonstrated in our group, which allows for 99.85% confidence in implanting a single donor [40]. This will result is a clean

stability diagram containing only one donor charge transition, and allow for the electrostatic tuning of the donor to the desired high-dipole regime [13].

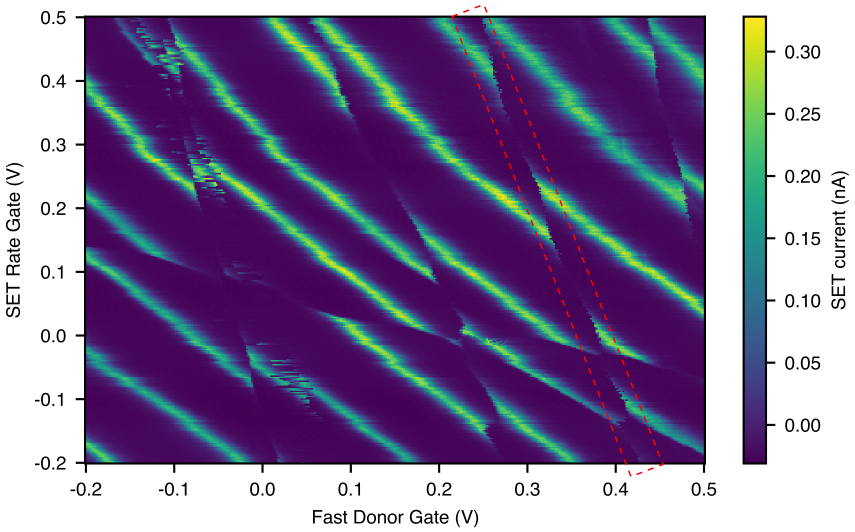

Supplementary Figure 13: **Charge stability diagram.** The donor charge transition of the flip-flop qubit (red dashed rectangle is surrounded by additional charge transitions due to other nearby implanted donors.

REFERENCES AND NOTES

1. A. J. Heinrich, W. D. Oliver, L. M. K. Vandersypen, A. Ardavan, R. Sessoli, D. Loss, A. B. Jayich, J. Fernandez-Rossier, A. Laucht, A. Morello, Quantum-coherent nanoscience, *Nat. Nanotechnol.* **16**, 1318–1329 (2021).

2. D. D. Awschalom, L. C. Bassett, A. S. Dzurak, E. L. Hu, J. R. Petta, Quantum spintronics: Engineering and manipulating atom-like spins in semiconductors, *Science* **339**, 1174–1179 (2013).

3. K. Saeedi, S. Simmons, J. Z. Salvail, P. Dluhy, H. Riemann, N. V. Abrosimov, P. Becker, H. J. Pohl, J. J. L. Morton, M. L. W. Thewalt, Room-temperature quantum bit storage exceeding 39 minutes using ionized donors in silicon-28, *Science* **342**, 830–833 (2013).

4. M. Zhong, M. P. Hedges, R. L. Ahlefeldt, J. G. Bartholomew, S. E. Beavan, S. M. Wittig, J. J. Longdell, M. J. Sellars, Optically addressable nuclear spins in a solid with a six-hour coherence time, *Nature* **517**, 177–180 (2015).

5. L. Vandersypen, H. Bluhm, J. S. Clarke, A. S. Dzurak, R. Ishihara, A. Morello, D. J. Reilly, L. R. Schreiber, M. Veldhorst, Interfacing spin qubits in quantum dots and donors—Hot, dense, and coherent. *npj Quantum Inf.* **3**, 34 (2017).

6. K. C. Nowack, F. Koppens, Y. V. Nazarov, L. Vandersypen, Coherent control of a single electron spin with electric fields. *Science* **318**, 1430–1433 (2007).

7. M. Pioro-Ladriere, T. Obata, Y. Tokura, Y.-S. Shin, T. Kubo, K. Yoshida, T. Taniyama, S. Tarucha, Electrically driven single-electron spin resonance in a slanting zeeman field. *Nat. Phys.* **4**, 776–779 (2008).

8. P. Klimov, A. Falk, B. Buckley, D. Awschalom, Electrically driven spin resonance in silicon carbide color centers. *Phys. Rev. Lett.* **112**, 087601 (2014).

9. A. J. Sigillito, A. M. Tyryshkin, T. Schenkel, A. A. Houck, S. A. Lyon, All-electric control of donor nuclear spin qubits in silicon. *Nat. Nanotechnol.* **12**, 958–962 (2017).

10. K. Yang, W. Paul, S. H. Phark, P. Willke, Y. Bae, T. Choi, T. Esat, A. Ardavan, A. J. Heinrich, C. P. Lutz, Coherent spin manipulation of individual atoms on a surface. *Science* **366**, 509–512 (2019).

11. S. Thiele, F. Balestro, R. Ballou, S. Klyatskaya, M. Ruben, W. Wernsdorfer, Electrically driven nuclear spin resonance in single-molecule magnets. *Science* **344**, 1135–1138 (2014).

12. S. Asaad, V. Mourik, B. Joecker, M. A. I. Johnson, A. D. Baczewski, H. R. Firgau, M. T. Mądzik, V. Schmitt, J. J. Pla, F. E. Hudson, K. M. Itoh, J. C. McCallum, A. S. Dzurak, A. Laucht, A. Morello, Coherent electrical control of a single high-spin nucleus in silicon. *Nature* **579**, 205–209 (2020).

13. G. Tosi, F. A. Mohiyaddin, V. Schmitt, S. Tenberg, R. Rahman, G. Klimeck, A. Morello, Silicon quantum processor with robust long-distance qubit couplings. *Nat. Commun.* **8**, 450 (2017).

14. B. E. Kane, A silicon-based nuclear spin quantum computer. *Nature* **393**, 133–137 (1998).

15. A. Laucht, J. T. Muhonen, F. A. Mohiyaddin, R. Kalra, J. P. Dehollain, S. Freer, F. E. Hudson, M. Veldhorst, R. Rahman, G. Klimeck, K. M. Itoh, D. N. Jamieson, J. C. McCallum, A. S. Dzurak, A. Morello, Electrically controlling single-spin qubits in a continuous microwave field. *Sci. Adv.* **1**, e1500022 (2015).

16. J. Mansir, P. Conti, Z. Zeng, J. J. Pla, P. Bertet, M. W. Swift, C. G. van de Walle, M. L. W. Thewalt, B. Sklenard, Y. M. Niquet, J. J. L. Morton, Linear hyperfine tuning of donor spins in silicon using hydrostatic strain. *Phys. Rev. Lett.* **120**, 167701 (2018).

17. J. J. Pla, K. Y. Tan, J. P. Dehollain, W. H. Lim, J. J. L. Morton, D. N. Jamieson, A. S. Dzurak, A. Morello, A single-atom electron spin qubit in silicon. *Nature* **489**, 541–545 (2012).

18. J. J. Pla, K. Y. Tan, J. P. Dehollain, W. H. Lim, J. J. L. Morton, F. A. Zwanenburg, D. N. Jamieson, A. S. Dzurak, A. Morello, High-fidelity readout and control of a nuclear spin qubit in silicon. *Nature* **496**, 334–338 (2013).

19. J. Dehollain, J. J. Pla, E. Siew, K. Y. Tan, A. S. Dzurak, A. Morello, Nanoscale broadband transmission lines for spin qubit control. *Nanotechnology* **24**, 015202 (2012).

20. G. Pica, G. Wolfowicz, M. Urdampilleta, M. L. W. Thewalt, H. Riemann, N. V. Abrosimov, P. Becker, H. J. Pohl, J. J. L. Morton, R. N. Bhatt, S. A. Lyon, B. W. Lovett, Hyperfine Stark effect of shallow donors in silicon. *Phys. Rev. B* **90**, 195204 (2014).

21. A. Morello, J. J. Pla, F. A. Zwanenburg, K. W. Chan, K. Y. Tan, H. Huebl, M. Möttönen, C. D. Nugroho, C. Yang, J. A. van Donkelaar, A. D. C. Alves, D. N. Jamieson, C. C. Escott, L. C. L. Hollenberg, R. G. Clark, A. S. Dzurak, Single-shot readout of an electron spin in silicon. *Nature* **467**, 687–691 (2010).

22. A. Laucht, R. Kalra, J. T. Muhonen, J. P. Dehollain, F. A. Mohiyaddin, F. Hudson, J. C. McCallum, D. N. Jamieson, A. S. Dzurak, A. Morello, High-fidelity adiabatic inversion of a ^{31}P electron spin qubit in natural silicon. *Appl. Phys. Lett.* **104**, 092115 (2014).

23. G. Feher, Electron spin resonance experiments on donors in silicon. I. Electronic structure of donors by the electron nuclear double resonance technique. *Phys. Rev.* **114**, 1219–1244 (1959).

24. K. Takeda, J. Yoneda, T. Otsuka, T. Nakajima, M. R. Delbecq, G. Allison, Y. Hoshi, N. Usami, K. M. Itoh, S. Oda, T. Kodera, S. Tarucha, Optimized electrical control of a Si/SiGe spin qubit in the presence of an induced frequency shift. *npj Quantum Inf.* **4**, 54 (2018).

25. S. Freer, S. Simmons, A. Laucht, J. T. Muhonen, J. P. Dehollain, R. Kalra, F. A. Mohiyaddin, F. E. Hudson, K. M. Itoh, J. C. McCallum, D. N. Jamieson, A. S. Dzurak, A. Morello, A single-atom quantum memory in silicon. *Quantum Sci. Technol.* **2**, 015009 (2017).

26. T. Watson, S. G. J. Philips, E. Kawakami, D. R. Ward, P. Scarlino, M. Veldhorst, D. E. Savage, M. G. Lagally, M. Friesen, S. N. Coppersmith, M. A. Eriksson, L. M. K. Vandersypen, A programmable two-qubit quantum processor in silicon. *Nature* **555**, 633–637 (2018).

27. A. Zwerver, T. Krähenmann, T. F. Watson, L. Lampert, H. C. George, R. Pillarisetty, S. A. Bojarski, P. Amin, S. V. Amitonov, J. M. Boter, R. Caudillo, D. Correas-Serrano, J. P. Dehollain, G. Droulers, E. M. Henry, R. Kotlyar, M. Lodari, F. Lüthi, D. J. Michalak, B. K. Mueller, S. Neyens, J. Roberts, N. Samkharadze, G. Zheng, O. K. Zietz, G. Scappucci, M. Veldhorst, L. M. K. Vandersypen, J. S. Clarke, Qubits made by advanced semiconductor manufacturing. *Nat. Electron.* **5**, 184–190 (2022).

28. E. Nielsen, J. K. Gamble, K. Rudinger, T. Scholten, K. Young, R. Blume-Kohout, Gate set tomography. *Quantum* **5**, 557 (2021).

29. E. Magesan, J. M. Gambetta, J. Emerson, Scalable and robust randomized benchmarking of quantum processes. *Phys. Rev. Lett.* **106**, 180504 (2011).

30. J. Järvinen, J. Ahokas, S. Sheludyakov, O. Vainio, L. Lehtonen, S. Vasiliev, D. Zvezdov, Y. Fujii, S. Mitsudo, T. Mizusaki, M. Gwak, S. G. Lee, S. Lee, L. Vlasenko, Efficient dynamic nuclear polarization of phosphorus in silicon in strong magnetic fields and at low temperatures. *Phys. Rev. B* **90**, 214401 (2014).

31. D. McCamey, J. Van Tol, G. Morley, C. Boehme, Fast nuclear spin hyperpolarization of phosphorus in silicon. *Phys. Rev. Lett.* **102**, 027601 (2009).

32. A. Yang, M. Steger, T. Sekiguchi, M. L. W. Thewalt, T. D. Ladd, K. M. Itoh, H. Riemann, N. V. Abrosimov, P. Becker, H. J. Pohl, Simultaneous subsecond hyperpolarization of the nuclear and electron spins of phosphorus in silicon by optical pumping of exciton transitions. *Phys. Rev. Lett.* **102**, 257401 (2009).

33. T. Sekiguchi, M. Steger, K. Saeedi, M. L. W. Thewalt, H. Riemann, N. V. Abrosimov, N. Nötzel, Hyperfine structure and nuclear hyperpolarization observed in the bound exciton luminescence of Bi donors in natural Si. *Phys. Rev. Lett.* **104**, 137402 (2010).

34. P. Gumann, O. Patange, C. Ramanathan, H. Haas, O. Moussa, M. L. W. Thewalt, H. Riemann, N. V. Abrosimov, P. Becker, H. J. Pohl, K. M. Itoh, D. G. Cory, Inductive

measurement of optically hyperpolarized phosphorous donor nuclei in an isotopically enriched silicon-28 crystal. *Phys. Rev. Lett.* **113**, 267604 (2014).

35. F. Hoehne, L. Dreher, D. P. Franke, M. Stutzmann, L. S. Vlasenko, K. M. Itoh, M. S. Brandt, Submillisecond hyperpolarization of nuclear spins in silicon. *Phys. Rev. Lett.* **114**, 117602 (2015).

36. G. W. Morley, M. Warner, A. M. Stoneham, P. T. Greenland, J. van Tol, C. W. M. Kay, G. Aeppli, The initialization and manipulation of quantum information stored in silicon by bismuth dopants. *Nat. Mater.* **9**, 725–729 (2010).

37. A. M. Tyryshkin, S. Tojo, J. J. L. Morton, H. Riemann, N. V. Abrosimov, P. Becker, H. J. Pohl, T. Schenkel, M. L. W. Thewalt, K. M. Itoh, S. A. Lyon, Electron spin coherence exceeding seconds in high-purity silicon. *Nat. Mater.* **11**, 143–147 (2012).

38. R. Deshpande, "Nuclear spin dynamics under above-bandgap optical pumping in silicon," thesis, University of Waterloo, Canada (2020).

39. F. A. Calderon-Vargas, E. Barnes, S. E. Economou, Fast high-fidelity single-qubit gates for flip-flop qubits in silicon. arXiv:2101.11592 (2021).

40. A. M. Jakob, S. G. Robson, V. Schmitt, V. Mourik, M. Posselt, D. Spemann, B. C. Johnson, H. R. Firgau, E. Mayes, J. C. McCallum, A. Morello, D. N. Jamieson, Deterministic shallow dopant implantation in silicon with detection confidence upper-bound to 99.85% by ion-solid interactions (Adv. Mater. 3/2022). *Adv. Mater.* **34**, 2270022 (2022).

41. M. T. Mądzik, S. Asaad, A. Youssry, B. Joecker, K. M. Rudinger, E. Nielsen, K. C. Young, T. J. Proctor, A. D. Baczewski, A. Laucht, V. Schmitt, F. E. Hudson, K. M. Itoh, A. M. Jakob, B. C. Johnson, D. N. Jamieson, A. S. Dzurak, C. Ferrie, R. Blume-Kohout, A. Morello, Precision tomography of a three-qubit donor quantum processor in silicon. *Nature* **601**, 348–353 (2022).

42. L. Fricke, S. J. Hile, L. Kranz, Y. Chung, Y. He, P. Pakkiam, M. G. House, J. G. Keizer, M. Y. Simmons, Coherent control of a donor-molecule electron spin qubit in silicon. *Nat. Commun.* **12**, 3323 (2021).

43. K. Yang, P. Willke, Y. Bae, A. Ferrón, J. L. Lado, A. Ardavan, J. Fernández-Rossier, A. J. Heinrich, C. P. Lutz, Electrically controlled nuclear polarization of individual atoms. *Nat. Nanotechnol.* **13**, 1120–1125 (2018).

44. C. M. Gilardoni, I. Ion, F. Hendriks, M. Trupke, C. H. van der Wal, Hyperfine-mediated transitions between electronic spin-1/2 levels of transition metal defects in SiC. *New J. Phys.* **23**, 083010 (2021).

45. A. L. Falk, P. V. Klimov, B. B. Buckley, V. Ivády, I. A. Abrikosov, G. Calusine, W. F. Koehl, Á. Gali, D. D. Awschalom, Electrically and mechanically tunable electron spins in silicon carbide color centers. *Phys. Rev. Lett.* **112**, 187601 (2014).

46. J. Liu, J. Mrozek, A. Ullah, Y. Duan, J. J. Baldoví, E. Coronado, A. Gaita-Ariño, A. Ardavan, Quantum coherent spin–electric control in a molecular nanomagnet at clock transitions. *Nat. Phys.* **17**, 1205–1209 (2021).

47. The Stopping and Range of Ions in Matter software.

48. J. H. Nielsen, W. H. Nielsen, M. Astafev, A. C. Johnson, D. Vogel, S. Chatoor, G. Ungaretti, A. M. Smiles, S. Pauka, P. Eendebak, Q. Saevar, P. Een-debak, R. van Gulik, N. Pearson, damazter, A. Corna, S. Droege, damazter2, T. Larsen, A. Geller, euchas, V. Hartong, S. Asaad, C. Granade, L. Drmi´c, S. Borghardt, Qcodes/qcodes: Qcodes 0.2.1 (2019).

49. S. Asaad, M. Johnson, Silq measurement software (2017).

50. J. M. Elzerman, R. Hanson, L. H. Willems van Beveren, B. Witkamp, L. M. K. Vandersypen, L. P. Kouwenhoven, Single-shot read-out of an individual electron spin in a quantum dot. *Nature* **430**, 431–435 (2004).

51. A. Morello, C. C. Escott, H. Huebl, L. H. Willems van Beveren, L. C. L. Hollenberg, D. N. Jamieson, A. S. Dzurak, R. G. Clark, Architecture for high-sensitivity single-shot readout and control of the electron spin of individual donors in silicon. *Phys. Rev. B* **80**, 081307 (2009).

52. M. A. Johnson, M. T. Mądzik, F. E. Hudson, K. M. Itoh, A. M. Jakob, D. N. Jamieson, A. Dzurak, A. Morello, Beating the thermal limit of qubit initialization with a Bayesian Maxwell's demon. *Phys. Rev. X* **12**, 041008 (2022).

53. V. B. Braginsky, F. Y. Khalili, Quantum nondemolition measurements: The route from toys to tools. *Rev. Mod. Phys.* **68**, 1–11 (1996).

54. P. Boross, G. Széchenyi, A. Pályi, Valley-enhanced fast relaxation of gate-controlled donor qubits in silicon, *Nanotechnology* **27**, 314002 (2016).

55. S. B. Tenberg, S. Asaad, M. T. Mądzik, M. A. I. Johnson, B. Joecker, A. Laucht, F. E. Hudson, K. M. Itoh, A. M. Jakob, B. C. Johnson, D. N. Jamieson, J. C. McCallum, A. S. Dzurak, R. Joynt, A. Morello, Electron spin relaxation of single phosphorus donors in metal-oxide-semiconductor nanoscale devices, *Phys. Rev. B* **99**, 205306 (2019).

56. W. Yang, W.-L. Ma, R.-B. Liu, Quantum many-body theory for electron spin decoherence in nanoscale nuclear spin baths, *Rep. Prog. Phys.* **80**, 016001 (2017).

57. M. T. Mądzik, T. D. Ladd, F. E. Hudson, K. M. Itoh, A. M. Jakob, B. C. Johnson, J. C. McCallum, D. N. Jamieson, A. S. Dzurak, A. Laucht, A. Morello, Controllable freezing of the nuclear spin bath in a single-atom spin qubit. *Sci. Adv.* **6**, eaba3442 (2020).

58. R. Blume-Kohout, J. K. Gamble, E. Nielsen, J. Mizrahi, J. D. Sterk, P. Maunz, Robust, self-consistent, closed-form tomography of quantum logic gates on a trapped ion qubit. arXiv:1310.4492 (2013).

59. D. Greenbaum, Introduction to quantum gate set tomography. arXiv:1509.02921 (2015).

60. R. Blume-Kohout, J. K. Gamble, E. Nielsen, K. Rudinger, J. Mizrahi, K. Fortier, P. Maunz, Demonstration of qubit operations below a rigorous fault tolerance threshold with gate set tomography. *Nat. Commun.* **8**, 14485 (2017).

61. pyGSTi A python implementation of gate set tomography, www.pygsti.info/.

62. R. Blume-Kohout, M. P. da Silva, E. Nielsen, T. Proctor, K. Rudinger, M. Sarovar, K. Young, A taxonomy of small markovian errors. *PRX Quantum* **3**, 020335 (2022).

63. J. Muhonen, A. Laucht, S. Simmons, J. P. Dehollain, R. Kalra, F. E. Hudson, S. Freer, K. M. Itoh, D. N. Jamieson, J. C. McCallum, Quantifying the quantum gate fidelity of single-atom spin qubits in silicon by randomized benchmarking. *J. Phys. Condens. Matter* **27**, 154205 (2015).

64. F. A. Mohiyaddin, R. Rahman, R. Kalra, G. Klimeck, L. C. L. Hollenberg, J. J. Pla, A. S. Dzurak, A. Morello, Noninvasive spatial metrology of single-atom devices. *Nano Lett.* **13**, 1903–1909 (2013).

65. D. Sivia, J. Skilling, *Data Analysis: A Bayesian Tutorial* (OUP Oxford, 2006).

66. F. A. Mohiyaddin, "Designing a large scale quantum computer with classical & quantum simulations," thesis, UNSW Sydney, Australia (2014).

67. L. Dreher, T. A. Hilker, A. Brandlmaier, S. T. B. Goennenwein, H. Huebl, M. Stutzmann, M. S. Brandt, Electroelastic hyperfine tuning of phosphorus donors in silicon, *Phys. Rev. Lett.* **106**, 037601 (2011).
